# HAAO‑derived quinolinic acid fuels FDPS‑dependent AR signaling and sensitizes prostate cancer to combination therapy

**DOI:** 10.1038/s41420-026-03203-x

**Published:** 2026-06-16

**Authors:** Hongchang Zhang, Tingting Feng, Mengxue Lv, Zhurui Shao, Changhao Gong, Ruiying Chen, Ruojia Zhang, Yong Hou, Jinxiang Han, Di Wang, Lin Wang

**Affiliations:** 1 College of Life Sciences, University of Chinese Academy of Sciences, Beijing, China; 2 BGI Research, Beijing, China; 3 Biomedical Sciences College, Shandong Medicinal Biotechnology Centre, Shandong First Medical University & Shandong Academy of Medical Sciences, Jinan, China

**Keywords:** Cancer metabolism, Tumour heterogeneity, Cancer therapy

## Abstract

Emerging evidence highlights that metabolic reprogramming profoundly shapes the tumor microenvironment and immune evasion in prostate cancer. However, the functional role and mechanisms of tryptophan metabolism in prostate cancer progression remain unclear. Through single-cell transcriptomic analysis, we identified one tumor cell subtype characterized by high expression of 3-hydroxyanthranilate 3,4-dioxygenase (HAAO) and enhanced kynurenine pathway activity. This subpopulation leads to the accumulation of quinolinic acid (QA), a metabolic intermediate that could activate the mevalonate (MVA) pathway. Mechanistically, QA directly binds to and stabilizes farnesyl diphosphate synthase (FDPS), a key MVA pathway enzyme, thereby enhancing cholesterol biosynthesis and fueling androgen receptor (AR)-driven transcriptional programs. This HAAO/QA-FDPS axis establishes a metabolic crosstalk that links tryptophan catabolism to lipid metabolism, sustaining prostate tumor progression. Furthermore, an integrated prognostic model incorporating this pathway signatures outperforms other clinical variables alone, and HAAO-high tumors exhibit heightened sensitivity to combined inhibition of the kynurenine and AR pathways. Our study unveils a novel metabolic vulnerability in prostate cancer and provides a mechanistic rationale for targeting the HAAO/QA-FDPS axis for therapy.

**Figure:**
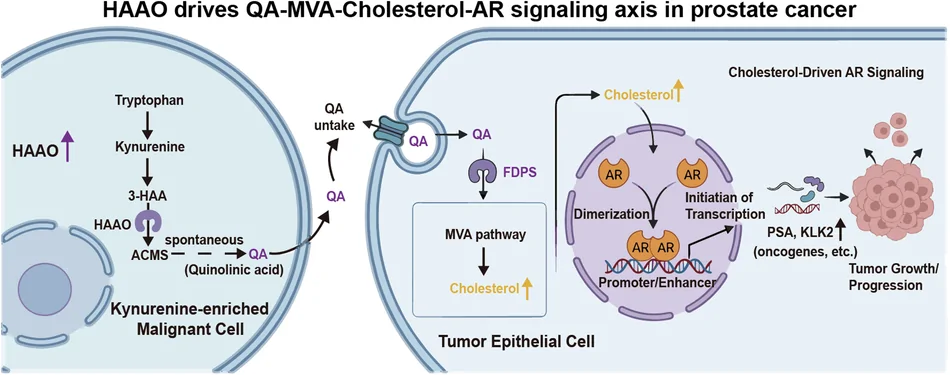

## Introduction

Prostate cancer is one of the most prevalent malignancies in men and exhibits remarkable molecular and metabolic heterogeneity [1]. Unlike many other solid tumors that rely heavily on the Warburg effect (anaerobic glycolysis), prostate cancer exhibits a distinct metabolic phenotype. While glucose metabolism and lactate production certainly contribute to invasion and immune evasion [2], prostate cancer cells show a unique and profound dependence on lipid and amino acid metabolism to fuel their proliferation and survival [3, 4]. Specifically, the reprogramming of these two metabolic pillars is now recognized not merely as a bystander effect, but as a driving force of tumorigenesis and drug resistance

Lipid metabolism, particularly the mevalonate (MVA) pathway, is central to prostate cancer progression. The MVA pathway is responsible for the de novo synthesis of cholesterol and isoprenoids, which serve as obligate precursors for androgen biosynthesis and protein prenylation, thereby sustaining androgen receptor (AR) signaling even under castrate conditions [5, 6]. Crucially, cholesterol serves as the obligate precursor for androgen biosynthesis, sustaining AR signaling—the primary driver of prostate cancer—even under castrate conditions [6]. Consequently, enzymes such as HMG-CoA reductase (HMGCR) and farnesyl diphosphate synthase (FDPS) are frequently upregulated in advanced disease. While inhibitors like statins have shown promise, clinical outcomes remain inconsistent, suggesting that tumor cells possess adaptive mechanisms to sustain MVA activity through compensatory metabolic rewiring [7, 8].

Parallel to lipid dysregulation, prostate cancer cells exhibit an insatiable demand for amino acids [9, 10]. Beyond the well-characterized roles of glutamine and leucine in fueling the TCA cycle and mTOR signaling [11, 12], tryptophan metabolism has emerged as a critical yet underappreciated frontier [12]. Tryptophan is primarily catabolized via the kynurenine pathway, a process historically viewed through the lens of immune suppression, where metabolites induce T-cell exhaustion and regulatory T-cell expansion in the tumor microenvironment [13, 14]. However, emerging metabolomic evidence indicates that tryptophan-derived metabolites are significantly elevated in prostate cancer tissues and serum [14, 15], hinting that the kynurenine pathway may exert direct oncogenic effects on epithelial cells beyond its immunomodulatory role.

Despite the established importance of both the MVA pathway (lipids) and the kynurenine pathway (amino acids), these metabolic programs have traditionally been studied in isolation. A critical knowledge gap remains: does a crosstalk exist where amino acid catabolism directly fuels lipid biosynthetic machinery? Understanding how these distinct metabolic networks integrate is essential, particularly in the context of castration-resistant prostate cancer (CRPC), where metabolic plasticity often drives therapy failure.

In this study, we employed single-cell RNA sequencing (scRNA-seq) to unravel a novel metabolic interplay linking tryptophan catabolism to cholesterol synthesis in prostate cancer progression. We identified a specific epithelial subpopulation characterized by high expression of 3-hydroxyanthranilate 3,4-dioxygenase (HAAO). We demonstrate that this subpopulation produces quinolinic acid (QA), a metabolite that acts as a paracrine signal to stabilize FDPS and augment MVA pathway activity in neighboring tumor cells. This previously unrecognized “kynurenine–mevalonate” axis promotes AR signaling and tumor progression, providing both a mechanistic explanation for metabolic resilience in prostate cancer and a new therapeutic vulnerability targeting the intersection of amino acid and lipid metabolism.

## Results

### scRNA-seq data identifies a kynurenine-high tumor subpopulation driven by HAAO in prostate cancer

**Fig. 1:**
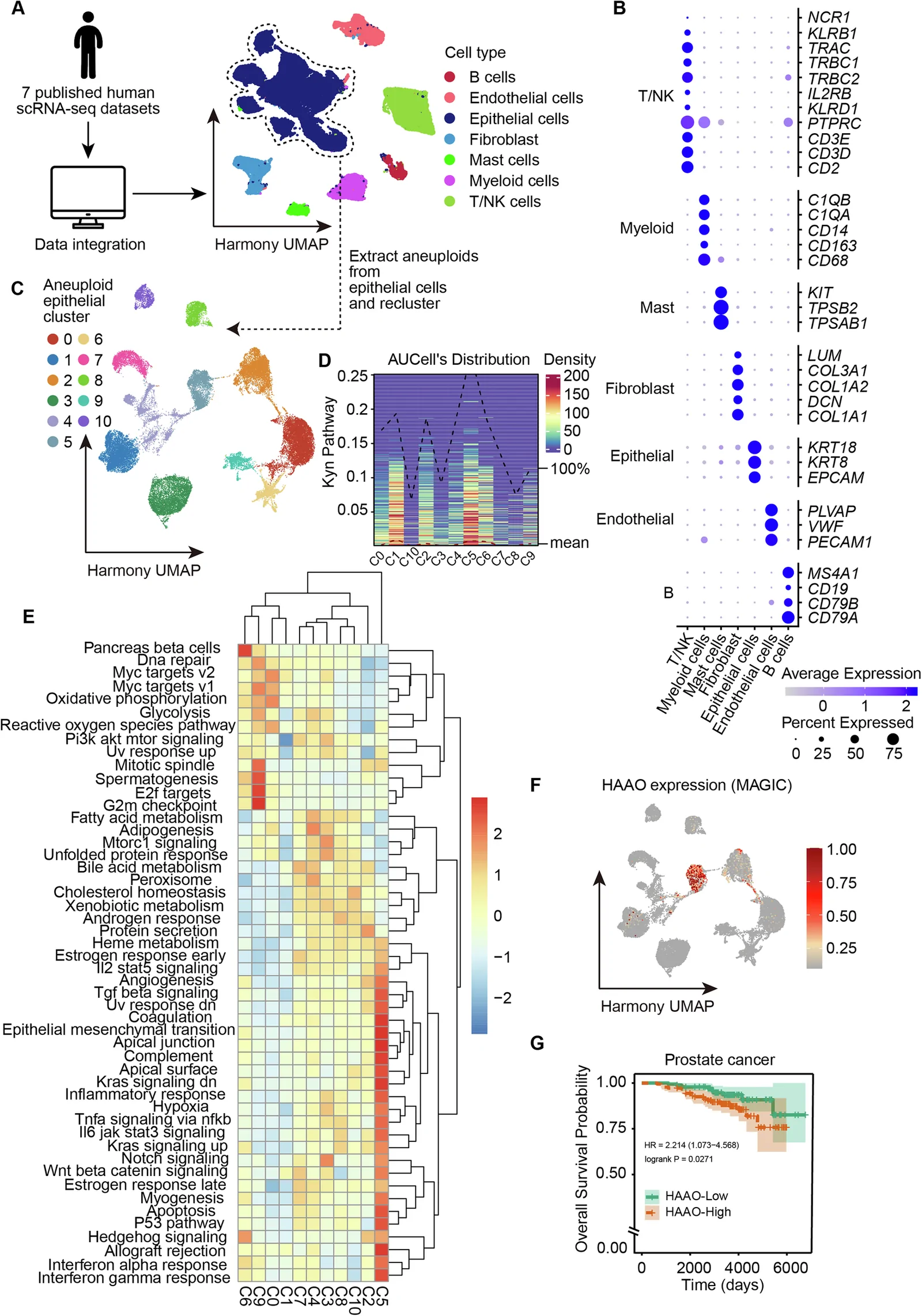
Kynurenine metabolism is correlated with the malignant progression of human Prostate cancer. **A** Integrated analysis of 123,986 cells from 7 published human scRNA-seq datasets. **B** Bubble heatmap showing expression levels of canonical markers in prostate cancer tissues. Dot size indicates fraction of expressing cells, colored based on average normalized expression levels. **C** UMAP plot showing the aneuploid epithelial cells. **D** Strip heatmap showing distributions of AUCell scores among aneuploid epithelial cells from 11 subclusters. **E** Heatmap plot showing hallmark pathway differences among aneuploid epithelial from 11 subclusters. **F** UMAP plot showing the expression of HAAO across various aneuploid epithelial cell types. **G** Kaplan–Meier analysis showing the association of HAAO expression with distant overall survival in prostate cancer patients.

To delineate the metabolic heterogeneity within tumor epithelial cells of prostate cancer, we integrated seven published single-cell RNA sequencing (scRNA-seq) datasets encompassing 38 prostate cancer patients, the total cells number is 123 986 (Fig. S1A). Based on canonical marker gene expression, we identified distinct cell populations, including B cells, endothelial cells, epithelial cells, fibroblasts, mast cells, myeloid cells, and T/NK cells (Fig. 1A, B). Next, we used CopyKAT to identify aneuploid epithelial cells as malignant tumor cells and then applied cNMF for dimensionality reduction and clustering, which divided the tumor epithelial compartment into 11 subclusters (Figs. 1C and S1B). Through scoring gene sets from different branches of the tryptophan metabolic network, we found the kynurenine pathway is the dominant route of tryptophan metabolism in prostate cancer (Fig. S1C).

To identify tumor subpopulations associated with tryptophan metabolism, we performed kynurenine pathway activity scoring with kynurenine-related genes across clusters. The results showed that the kynurenine pathway was enriched in cluster C5 (Fig. 1D). Differential gene expression analysis further revealed that cluster C5 was characterized by upregulation of basal epithelial markers, including KRT5 and KRT7, suggesting a basal-like and epithelial plasticity-associated state (Figure S1D). Hallmark pathway enrichment analysis further demonstrated that cluster C5 upregulated tumor progression associated pathways, including epithelial–mesenchymal transition (EMT) and p53 signaling (Fig. 1E). KEGG analysis also showed that cluster C5 was enriched in biological processes related to intermediate filaments, suggesting stronger resistance to mechanical stress and enhanced cell migration (Fig. S1E). In addition, module score analysis showed that both stemness and EMT scores were significantly higher in cluster C5 compared with other epithelial subpopulations (Fig. S1F). The results also showed that cluster C5 was present across the most datasets, indicating that cluster C5 was a reproducible and robust tumor subpopulation and may represent a conserved cellular state in prostate cancer (Fig. S1G). Further characterization of the cluster C5 revealed elevated expression of HAAO, a key catalytic enzyme in the kynurenine pathway (Figs. 1F and S1H). Notably, high HAAO expression was associated with poorer prognosis in prostate cancer patients (Fig. 1G). Collectively, these results indicate that dysregulated kynurenine metabolism, characterized by a distinct HAAO-high tumor subpopulation, was linked to aggressive features and adverse clinical outcomes in prostate cancer.

### HAAO drives prostate cancer progression in vivo and in vitro

**Fig. 2:**
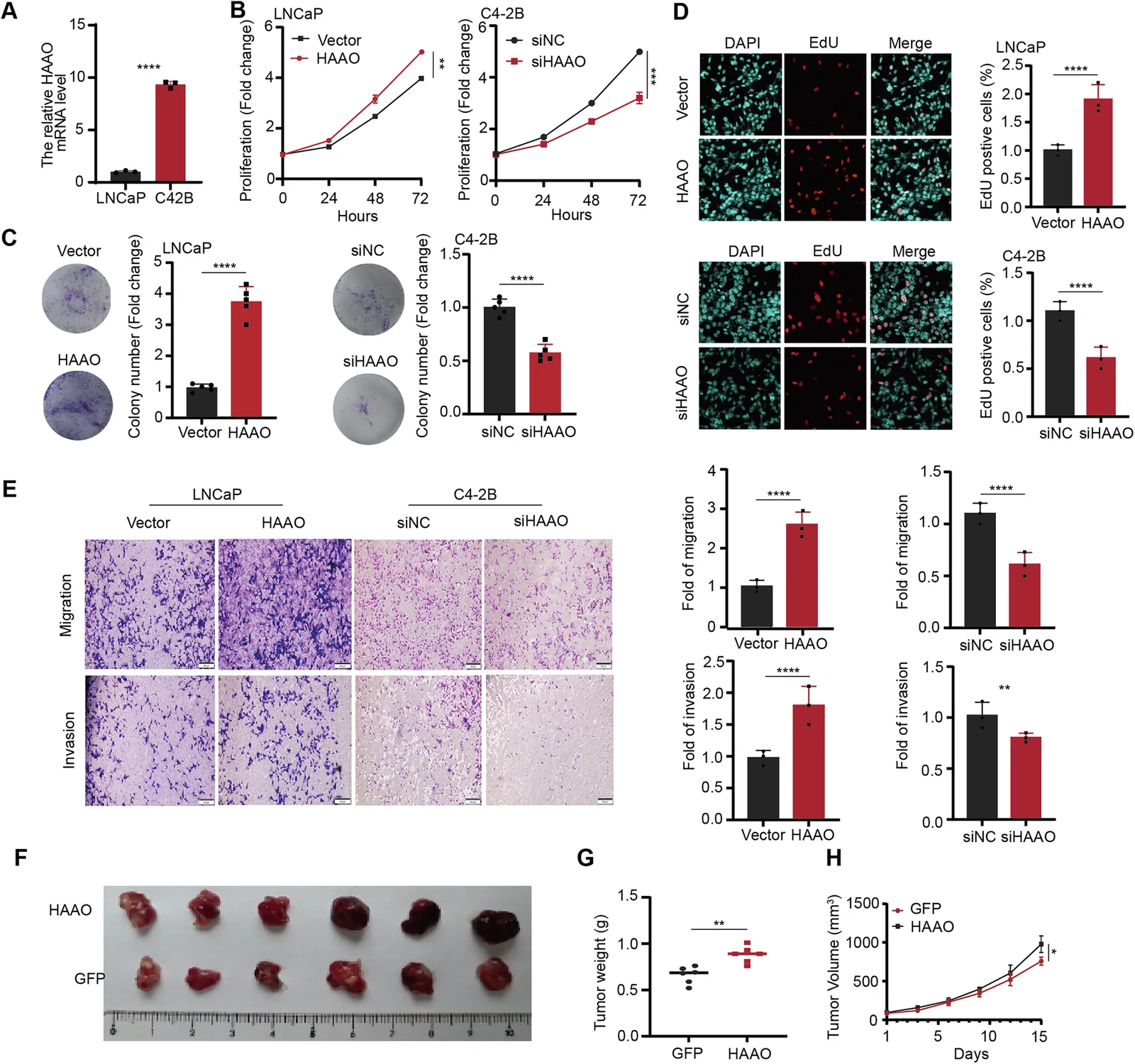
HAAO drives prostate cancer progression in vivo and in vitro. **A** Relative mRNA levels of HAAO in LNCaP and C4-2B cells. **B** CCK-8 assay of OE-HAAO in LNCaP and siHAAO in C4-2B cells. Data are presented as the mean ± SD. Unpaired two-tailed Student’s *t*-test used for comparison. ∗∗*p* < 0.01, ∗∗∗*p* < 0.001. **C** Colony formation assay of OE-HAAO in LNCaP and siHAAO in C4-2B cells. Data are presented as the mean ± SD. Unpaired two-tailed Student’s *t*-test used for comparison. ∗∗∗∗*p* < 0.0001. **D** EdU assay of OE-HAAO in LNCaP and siHAAO in C4-2B cells. Nuclei were stained with DAPI. Data are presented as the mean ± SD. Unpaired two-tailed Student’s *t*-test used for comparison. ∗∗∗∗*p* < 0.0001. **E** Transwell assays showing migration and invasion ability of OE-HAAO in LNCaP and siHAAO in C4-2B cells. Data are presented as the mean ± SD. Unpaired two-tailed Student’s *t*-test used for comparison. ∗∗*p* < 0.01, ∗∗∗∗*p* < 0.0001. Tumor images (**F**), endpoint weights (**G**), and volumes (**H**) of GFP vs HAAO-OE LNCaP allografts in wild-type BALB/c mice. *n* = 6 per group for all panels. For **G**, data are presented as mean ± SD, unpaired two-tailed Student’s t test. ** < 0.01. For (**H**), data are presented as mean ± SE, and endpoint data were analyzed using unpaired two-tailed Student’s *t*-test. * < 0.05.

To investigate the function role of HAAO in prostate cancer, we first assessed the baseline expression of HAAO in human prostate cancer cell lines LNCaP and C4-2B. The results showed that HAAO expression was over 10 times higher in C4-2B cells compared to LNCaP cells (Figs. 2A and S2A). We further evaluated the biological role of HAAO in prostate cancer cells by silencing HAAO in C4-2B and overexpress it in LNCaP (Figs. S2B–S2D). CCK-8 and colony formation assays showed that HAAO overexpression significantly enhanced the proliferation of LNCaP cells, whereas HAAO knockdown markedly suppressed proliferation in C4-2B cells (Fig. 2B, C). The result of EdU assay is consistent with these findings (Fig. 2D). These results further confirm that HAAO positively regulates prostate cancer cell growth. Furthermore, Transwell assays showed that HAAO overexpression promoted, while its silencing suppressed, cell migration and invasion (Fig. 2E). In vivo, HAAO-overexpressing LNCaP cells also demonstrated significantly accelerated tumor growth in nude mice compared to controls (Fig. 2F–H). These results reveal that HAAO promotes the proliferation, migration, and invasion abilities of prostate cancer cells.

Previous multi-omics studies have identified HAAO as a putative aneuploidy-associated driver gene, whose expression correlates with chromosomal instability and tumor progression [16], consistent with our experimental findings. Taken together, these results establish HAAO as a key functional driver that enhances proliferative, migratory, and invasive capacities in prostate cancer cells and promotes tumor growth in vivo.

### HAAO-mediated malignant progression of prostate cancer is executed through the accumulation of quinolinic acid (QA)

**Fig. 3:**
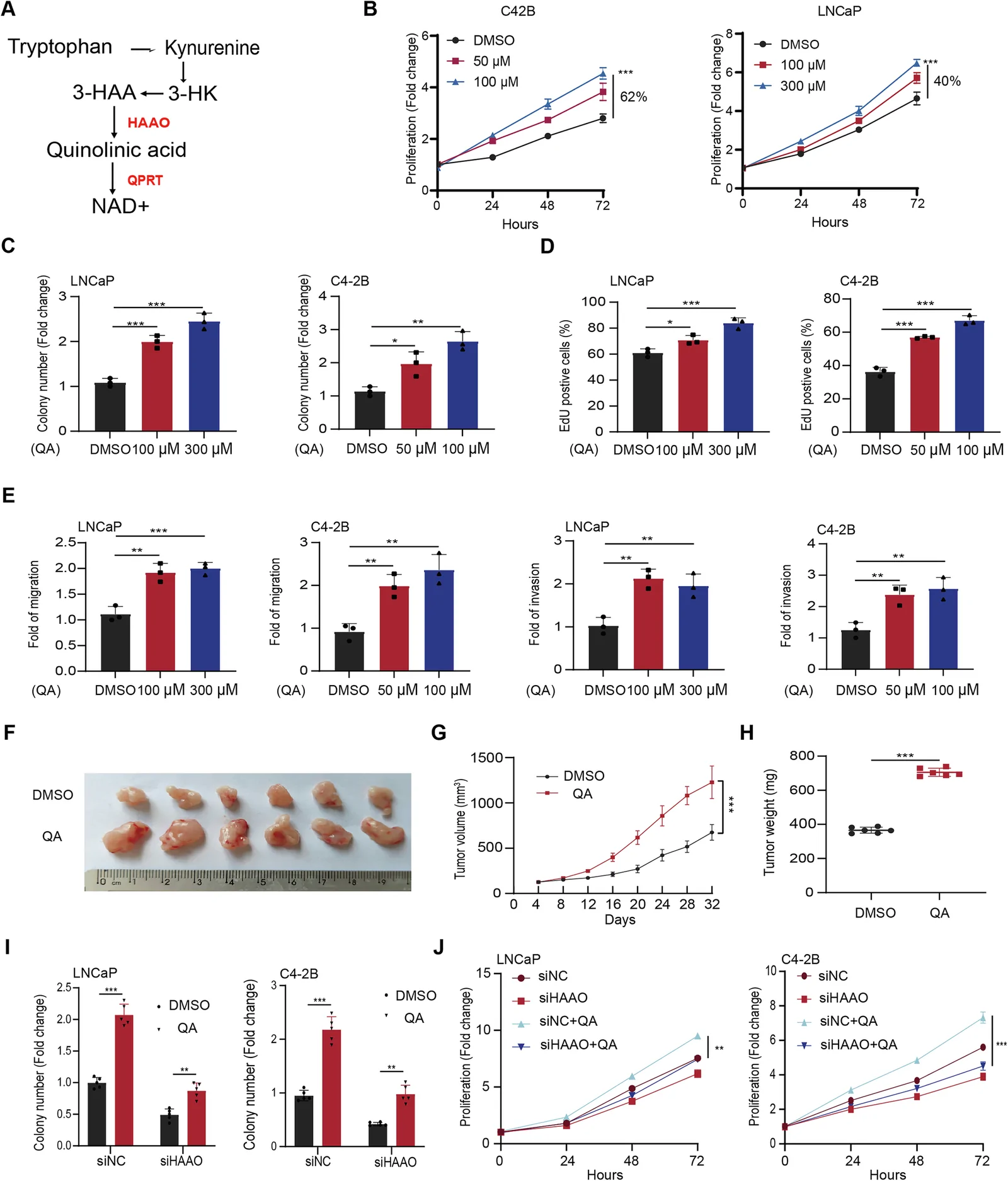
Kynurenine metabolism-mediated malignant progression of prostate cancer is associated with QA accumulating in the tumor microenvironment. **A** Kynurenine metabolic pathway in human prostate cancer cells. **B** CCK-8 assay of LNCaP and C4-2B cells treated with different QA concentrations. Data are presented as the mean ± SD. ∗∗∗*p* < 0.001. **C** Colony formation assay of LNCaP and C4-2B cells treated with different QA concentrations. Data are presented as the mean ± SD. Unpaired two-tailed Student’s *t*-test used for comparison. ∗*p* < 0.05, ∗∗*p* < 0.01, ∗∗∗*p* < 0.001. **D** EdU assay of LNCaP and C4-2B cells treated with different QA concentrations. Nuclei were stained with DAPI. Data are presented as the mean ± SD. Unpaired two-tailed Student’s *t*-test used for comparison. ∗*p* < 0.05, ∗∗∗*p* < 0.001. **E** Transwell assay showing migration and invasion ability of LNCaP and C4-2B cells treated with different QA concentrations. Data are presented as the mean ± SD. Unpaired two-tailed Student’s *t*-test used for comparison. ∗∗*p* < 0.01, ∗∗∗*p* < 0.001. Tumor images (**F**), endpoint weights (**G**), and volumes (**H**) of LNCaP allografts in wild-type BALB/c mice treated with different QA concentrations. Mice received 100 µL intraperitoneal (i.p.) QA injections every 2 days, starting 4 days post-inoculation. *n* = 6 per group for all panels. For (**G**), data are presented as mean ± SD, and endpoint data were analyzed using one-way ANOVA followed by Dunnett’s test for multiple comparisons. For (**H**), data are presented as mean ± SE, one-way ANOVA followed by Dunnett’s test for multiple comparisons. ***p < 0.001. **I** Colony formation assay of siHAAO in LNCaP and C4-2B cells treated with or without QA. Data are presented as the mean ± SD. Unpaired two-tailed Student’s *t*-test used for comparison. ∗∗*p* < 0.01, ∗∗∗*p* < 0.001. **J** CCK-8 assay of siHAAO in LNCaP and C4-2B cells treated with or without QA. Data are presented as the mean ± SD. Unpaired two-tailed Student’s *t*-test used for comparison. ∗∗p < 0.01, ∗∗∗p < 0.001.

HAAO is a key enzyme in the kynurenine metabolic pathway that catalyzes the conversion of 3-hydroxyanthranilic acid (3-HAA) to QA, which is subsequently metabolized into NAD⁺ by quinolinate phosphoribosyltransferase (QPRT) (Fig. 3A). Multiple RNA-seq datasets revealed that QPRT expression was significantly downregulated in prostate tumor tissues compared with adjacent normal tissues, suggesting that dysregulated kynurenine metabolism may lead to QA accumulation within the tumor microenvironment (Fig. S3A).

To investigate the biological effects of QA on prostate cancer cells, we selected four human prostate cancer cell lines, including AR-negative (PC3 and DU145) and AR-positive (LNCaP and C4-2B) models. We first determined the non-cytotoxic concentrations of QA to exclude potential confounding effects of cell death (Fig. S3B). CCK-8 assays showed that treatment with a relatively low concentration of QA increased proliferation in AR-positive cell lines, with LNCaP and C4-2B cells exhibiting 40% and 62% increases, respectively (Fig. 3B). In contrast, AR-negative cell lines (PC3 and DU145) displayed only modest proliferative responses (23% and 16%, respectively), even when treated with a higher concentration of QA (Fig. S3C). This finding suggests that AR signaling status may modulate the biological response to QA in prostate cancer cells.

To further evaluate the phenotypic effects of QA on AR-positive prostate cancer cells, colony formation, EdU, and Transwell assays were performed. The results showed that QA treatment markedly enhanced proliferation, migration, and invasion in both LNCaP and C4-2B cell lines (Fig. 3C–E). Furthermore, QA significantly attenuated tunicamycin (TM) and thapsigargin (TG) induced apoptosis in prostate cancer cells, with a more pronounced effect in C4-2B than in LNCaP cells (Fig. S3D). Consistent with the in vitro findings, QA administration promoted tumor growth in LNCaP xenografts in mice (Fig. 3F–H). Collectively, these findings indicate that dysregulation of the kynurenine pathway leads to QA accumulation, which in turn facilitates prostate cancer progression.

To investigate whether QA mediates paracrine effects in the tumor microenvironment, we modulated HAAO expression in prostate cancer cell lines by overexpression in LNCaP cells and knockdown in C4-2B cells, and measured QA levels in conditioned media by ELISA. We found that HAAO expression regulated extracellular QA levels, with overexpression in LNCaP cells significantly increasing QA secretion, whereas HAAO knockdown in C4-2B cells markedly reduced QA levels in the supernatant (Fig. S3E). Importantly, QA supplementation effectively reversed the growth inhibition induced by HAAO knockdown (Fig. 3I, J), supporting a functional role of QA in mediating the tumor-promoting effects of HAAO. Taken together, these findings indicate that the tumor promoting function of HAAO is at least partially mediated through QA accumulation in the tumor microenvironment.

### QA activates the mevalonate (MVA) pathway to drive cholesterol-dependent AR signaling and prostate cancer progression

**Fig. 4:**
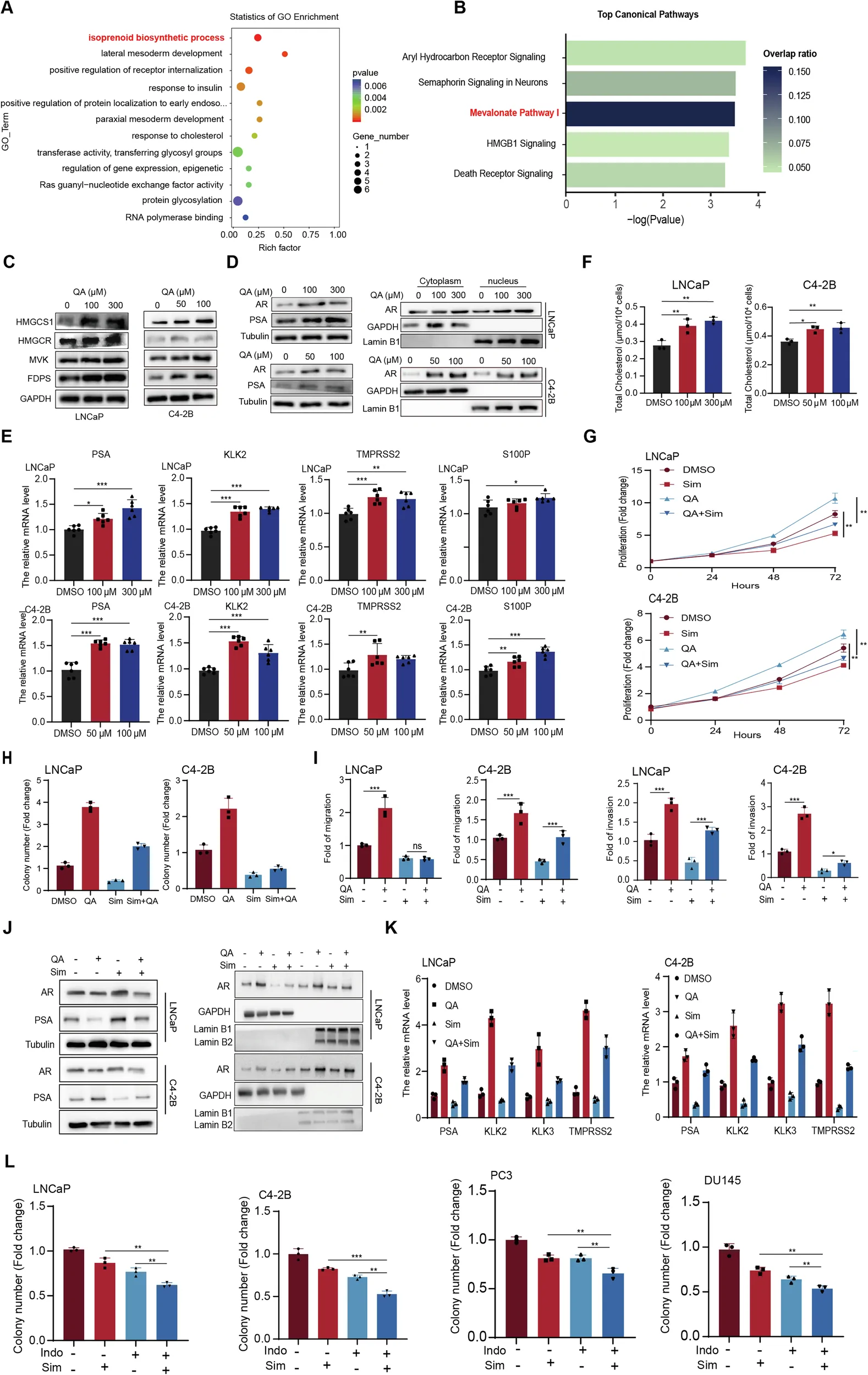
Quinolinic acid activates the MVA pathway to enhance AR-driven prostate cancer progression. **A** GO enrichment analysis showing the biological processes altered in LNCaP cells treated with QA. **B** IPA analysis showing the top canonical pathways in LNCaP cells treated with QA. **C** Western blot analysis showing the expression of MVA pathway proteins in LNCaP and C4-2B cells treated with different QA concentrations. **D** Bar plot showing the cholesterol levels in LNCaP and C4-2B cells treated with different QA concentrations. Data are presented as mean ± SD, and endpoint data were analyzed using one-way ANOVA followed by Dunnett’s test for multiple comparisons. ∗∗*p* < 0.01, ∗∗∗*p* < 0.001. **E** Western blot analysis showing AR signaling proteins and the nucleus vs cytoplasm distribution of AR in LNCaP and C4-2B cells treated with QA. **F** Bar plot showing the relative expression of AR signaling genes in LNCaP and C4-2B cells treated with different QA concentrations. **G** CCK-8 assay showing the viability of LNCaP and C4-2B cells under four treatment conditions: DMSO, QA alone, Sim alone, and QA plus Sim. Data are presented as the mean ± SD. ∗∗∗*p* < 0.001. Sim: simvastatin. **H** Colony formation assay of LNCaP and C4-2B cells under four treatment conditions: DMSO, QA alone, Sim alone, and QA plus Sim. Data are presented as the mean ± SD. Unpaired two-tailed Student’s *t*-test used for comparison. ∗∗∗*p* < 0.001. Sim: simvastatin. **I** Transwell assay showing migration and invasion ability of LNCaP and C4-2B cells treated with four treatment conditions: DMSO, QA alone, Sim alone, and QA plus Sim. Data are presented as the mean ± SD. Unpaired two-tailed Student’s *t*-test used for comparison. ∗*p* < 0.05, ∗∗∗*p* < 0.001. Sim: simvastatin. **J** Western blot analysis showing AR signaling proteins and the nucleus vs cytoplasm distribution of AR in LNCaP and C4-2B cells treated under four treatment conditions: DMSO, QA alone, Sim alone, and QA plus Sim. Sim: simvastatin. **(K** Bar plot showing the relative expression of AR signaling genes in LNCaP and C4-2B cells treated under four treatment conditions: DMSO, QA alone, Sim alone, and QA plus Sim. Data are presented as the mean ± SD. Unpaired two-tailed Student’s *t*-test used for comparison. ∗p < 0.05, ∗∗∗p < 0.001. Sim: simvastatin. **L** Colony formation assay of LNCaP, C4-2B cells, PC3, and DU145 under four treatment conditions: DMSO, Sim alone, Indo alone, and Indo plus Sim. Data are presented as the mean ± SD. Unpaired two-tailed Student’s *t*-test used for comparison. ∗∗*p* < 0.01, ∗∗∗*p* < 0.001. Sim: simvastatin, Indo: indoximod.

To investigate how QA accumulation influences tumor progression, we performed RNA-seq analysis on prostate cancer cell lines before and after QA treatment. GO analysis revealed that the differentially expressed genes (DEGs) after QA treatment were significantly enriched in biological processes such as isoprenoid biosynthetic process (Fig. 4A), Ingenuity Pathway Analysis (IPA) showed that QA treatment significantly affected pathways related to tumor progression, including the MVA Pathway I (Fig. 4B). Consistent with this observation, the MVA pathway represents the central metabolic route governing isoprenoid biosynthesis, indicating that QA induced transcriptional reprogramming converges on MVA-dependent isoprenoid production to potentially drive tumor progression.

To investigate whether QA modulates tumor metabolic pathways, we treated two prostate cancer cell lines, LNCaP and C4-2B, with QA and examined changes in the MVA pathway at both the protein and metabolite levels. Western blot analysis revealed that QA treatment led to significant upregulation of key enzymes in the MVA pathway, including HMGCS1, HMGCR, MVK, and FDPS (Fig. 4C), as well as proteins involved in downstream prenylation processes, such as RheB, RhoA, GGPS1, and Rap1A (Fig. S4A). Furthermore, targeted metabolomic profiling using LC-MS showed that QA treatment increased the intracellular levels of several MVA pathway derived metabolites, notably geranylgeranyl pyrophosphate (GGPP) and Cholesterol (Figs. 4D and S4B).

Given that cholesterol is the precursor for androgen biosynthesis and the MVA pathway is directly involved in cholesterol production, we investigated whether QA modulates androgen receptor (AR) signaling via the MVA pathway. In LNCaP and C4-2B cells, QA treatment led to a marked increase in AR and its canonical target gene PSA (KLK3) expression, along with enhanced nuclear translocation of AR (Fig. 4E), suggesting increased AR pathway activity. Expression of additional AR downstream targets (e.g., TMPRSS2) was also elevated upon QA exposure (Fig. 4F). Collectively, these findings demonstrate that QA enhances MVA pathway activity in prostate cancer cells, potentially contributing to tumor metabolic reprogramming through both transcriptional upregulation of rate-limiting enzymes and accumulation of downstream intermediates.

To further determine whether the tumor-promoting effects of QA are dependent on the MVA pathway, we treated prostate cancer cell lines LNCaP and C4-2B with QA in the presence or absence of simvastatin, a classical MVA pathway inhibitor. Functional assays, including CCK-8 and colony formation assays, demonstrated that QA significantly enhanced cell proliferation, whereas co-treatment with simvastatin effectively reversed this pro-proliferative effect (Fig. 4G, H). Similarly, QA promoted the migratory and invasive capacity of prostate cancer cells, but these phenotypes were significantly suppressed by simvastatin co-treatment (Fig. 4I). These findings indicate that the tumor-promoting activity of QA is dependent on an intact MVA pathway, and that simvastatin can antagonize QA-induced malignant phenotypes by blocking this metabolic axis. Importantly, co-treatment with simvastatin significantly reduced AR and PSA protein levels, suppressed AR nuclear translocation, and decreased expression of downstream AR-responsive genes. These results indicate that QA enhances AR signaling through activation of the MVA pathway, and that pharmacological inhibition of MVA metabolism can attenuate this effect (Fig. 4J, K). This highlights a critical metabolism–hormone signaling axis whereby QA promotes prostate cancer progression.

We confirmed that the kynurenine metabolite QA establishes a metabolic crosstalk with the MVA pathway. However, the precise mechanism by which QA regulates MVA activity remains unclear. Given that QA can be metabolized into NAD⁺, we hypothesized that QA might be taken up and converted by tumor cells to generate NAD⁺, which could serve as a cofactor for key enzymes in the MVA pathway, thereby enhancing its activity (Fig. S4C). To test this hypothesis, prostate cancer cells were treated with QA in the presence of a QPRT inhibitor, which blocks the conversion of QA to NAD⁺. Interestingly, QA treatment still led to a marked upregulation of MVA pathway activity, indicating that QA regulates the MVA pathway through an NAD⁺ independent mechanism to promote prostate cancer progression (Fig. S4D). These results demonstrate that QA activates the MVA pathway, leading to increased cholesterol synthesis and consequent potentiation of AR signaling, which establishes a metabolic-hormonal axis essential for QA-driven prostate cancer progression.

HAAO upregulation increases QA accumulation and drives activation of the MVA pathway. We next wonder whether simultaneous inhibition of these pathways could more effectively suppress prostate cancer progression. To address this question, we treated four human prostate cancer cells with inhibitors targeting the kynurenine pathway and MVA pathway, either alone or in combination. As shown in Fig. 4L, single-agent treatment suppressed cell proliferation, and this effect was further enhanced by the combination therapy, notably, AR-positive cell lines, such as LNCaP and C4-2B, exhibited greater sensitivity to the combination treatment compared with AR-negative cells.

### QA directly binds and stabilizes FDPS to sustain MVA pathway activity

**Fig. 5:**
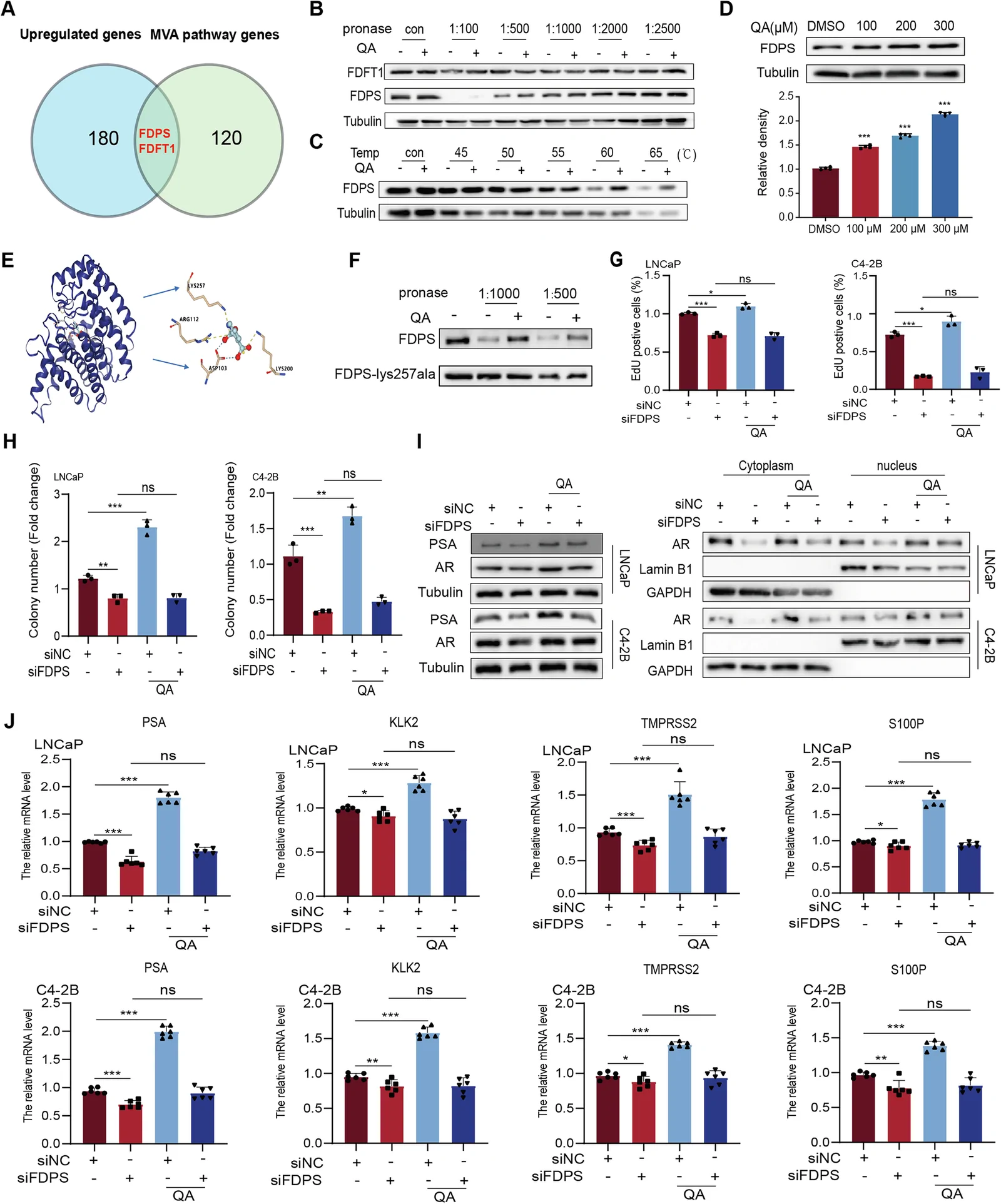
Quinolinic acid activates the MVA pathway by stabilizing FDPS. **A** Venn diagram showing the upregulated proteins in the MVA pathway after QA treatment in LNCaP cells. **B** DARTS assay performed in LNCaP and C4-2B cells treated with QA, showing the detected levels of FDPS and FDFT1 compared with untreated controls. **C** Cellular thermal shift assay in LNCaP and C4-2B cells treated without or with QA. **D** Western blot showing the expression levels of FDPS in LNCaP cells after treatment with different concentrations of QA. **E** Molecular docking model showing the potential binding site of QA on FDPS. **F** DARTS assay performed in LNCaP and C4-2B cells treated with QA, showing the detected levels of FDPS and FDPS-lys257ala compared with untreated controls. **G** EdU assay of LNCaP and C4-2B cells transfected with siNC or siFDPS in the presence of QA (*n* = 3). Nuclei were stained with DAPI. Data are presented as mean ± SD, and endpoint data were analyzed using one-way ANOVA followed by Dunnett’s test for multiple comparisons. ∗*p* < 0.05, ∗∗∗*p* < 0.001. **H** Colony formation assay of LNCaP and C4-2B cells transfected with siNC or siFDPS in the presence of QA (*n* = 3). Data are presented as mean ± SD, and endpoint data were analyzed using one-way ANOVA followed by Dunnett’s test for multiple comparisons. ∗*p* < 0.05, ∗∗∗*p* < 0.001. **I** Western blot analysis showing AR signaling proteins and the nucleus vs cytoplasm distribution of AR in LNCaP and C4-2B cells transfected with siNC or siFDPS in the presence of QA (*n* = 3). **J** Bar plot showing the relative expression of AR signaling genes in LNCaP and C4-2B cells transfected with siNC or siFDPS in the presence of QA (*n* = 3). Data are presented as mean ± SD, and endpoint data were analyzed using one-way ANOVA followed by Dunnett’s test for multiple comparisons. ∗*p* < 0.05, ∗∗*p* < 0.01, ∗∗∗*p* < 0.001.

To investigate how QA regulates MVA pathway activity, prostate cancer cell lines were treated with QA followed by mass spectrometry analysis (Fig. S5A). Interestingly, IPA analysis of the proteomic data revealed that QA treated prostate cancer cells were enriched for cholesterol biosynthesis pathways and showed activation of AR signaling. This observation was consistent with our previous RNA-seq findings and further supports a positive role of QA in sustaining cholesterol synthesis and AR pathway activity (Figs. S5B and S5C). The identified candidate molecules were intersected with genes involved in the MVA pathway, leading to the identification of FDPS and FDFT1 as potential targets of QA (Fig. 5A).

To validate direct interaction, we employed DARTS and CETSA assays. Both assays showed that QA treatment increased the resistance of FDPS to protease digestion and thermal denaturation, indicating enhanced structural stability and suggesting QA binding to FDPS (Fig. 5B, C). Furthermore, FDPS expression was positively correlated with the concentration of QA, suggesting a dose-dependent regulatory effect (Fig. 5D).

Next, we performed molecular docking analysis to predict potential QA-binding residues on FDPS. Four candidate interaction sites were identified (Fig. 5E). We then generated point-mutated FDPS constructs targeting these residues and evaluated their response to QA treatment using Western blot. The results showed that only mutation of lysine at position 257 (K257) abolished the protective effect of QA on FDPS stability (Fig. 5F). These results indicate that QA directly binds to FDPS at K257, stabilizing the protein and potentially enhancing MVA pathway activity in prostate cancer cells.

To further validate FDPS as a key molecule linking the kynurenine pathway to AR signaling, we silenced FDPS using siRNA and examined how this affected AR activation induced by exogenous QA. Functionally, EdU and colony formation assays demonstrated that FDPS knockdown significantly impaired the proliferative capacity of LNCaP and C4-2B cells, and markedly attenuated the growth promoting effects of QA (Fig. 5G, H). These results indicate that FDPS is required for QA-driven tumor cell proliferation. Moreover, compared with the control group, FDPS knockdown decreased the expression of AR pathway target proteins and genes and reduced AR nuclear translocation, indicating that FDPS supports AR pathway activation, when exogenous QA was added under FDPS-deficient conditions, QA was no longer able to activate AR signaling, further showing that FDPS is required for QA-driven AR pathway activation (Fig. 5I, J). These results demonstrate that FDPS is an essential mediator through which QA activates the MVA pathway and regulates AR signaling in prostate cancer cells.

### A clinically integrated machine-learning model based on pathways-associated genes improves prostate cancer prognosis prediction

**Fig. 6:**
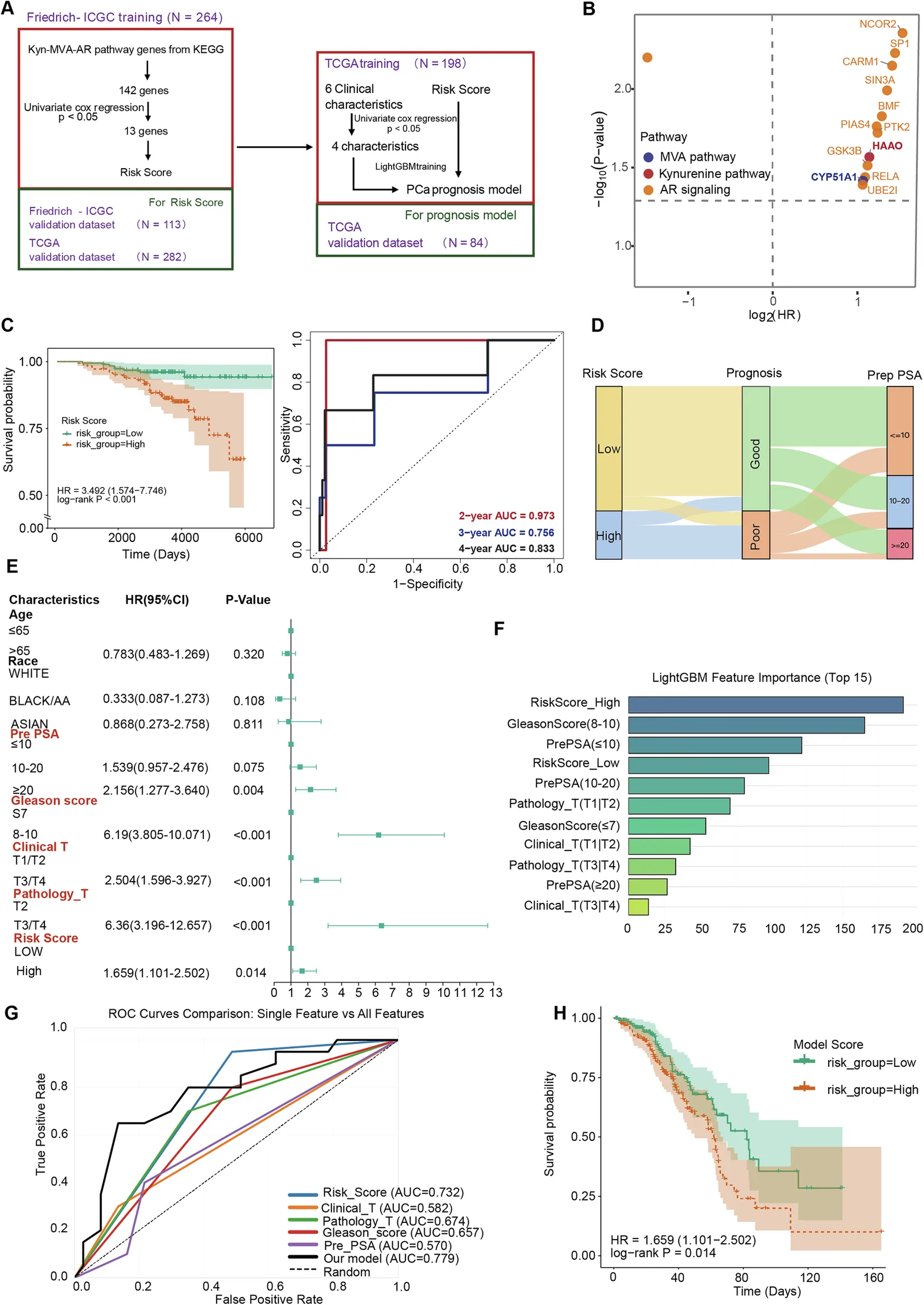
Pathway derived 13-gene signature and LightGBM integrated clinical characteristics model for prostate cancer prognosis prediction. **A** Workflow for the development and validation of the 13-gene signature and LightGBM integrated clinical characteristics model for prostate cancer prognosis prediction. **B** Volcano plot showing genes with *p*-values < 0.05 from univariate regression analysis across the three pathways: MVA, kynurenine pathway, and AR signaling. **C** Kaplan–Meier plot of high and low-risk group (left panel) and ROC curves (right panel) of the Risk_Score in the Friedrich-ICGC validation dataset, with a 2-year predictive power (red), a 3-year predictive power (blue), and a 4-year predictive power (black). **D** Sankey plot showing the overlap between high and low-risk groups defined by the risk score, preoperative PSA levels, and actual clinical outcomes in prostate cancer patients. **E** Forest plot showing the prognostic score for each clinical parameter in a multivariate Cox regression analysis. The middle points indicate the hazard ratios. The endpoints represent the lower or upper 95% confidence intervals. **F** Bar chart showing the LightGBM feature importance. **G** ROC curves comparing the predictive accuracy of the single feature versus all features. **H** Kaplan–Meier plots of high and low-risk group based on the integrated model in the TCGA validation dataset.

At present, reliable prognostic markers that can both predict outcome and guide treatment in prostate cancer are still limited in clinical practice. As shown in the workflow (Fig. 6A), we selected a total of 142 pathway genes from the kynurenine pathway, the MVA pathway, and the AR signaling pathway, and used univariate Cox regression to identify genes significantly associated with prognosis. This analysis yielded 13 survival-related genes (Fig. S6A), including the key kynurenine pathway enzyme HAAO (Fig. 6B). Using the expression levels of these 13 genes and their regression coefficients, we constructed a composite risk score. In the Friedrich and ICGC validation cohorts, patients in the high-risk group consistently exhibited significantly poorer survival than those in the low-risk group and time-dependent AUC analysis further demonstrated that the Risk score robustly identified patients with unfavorable 2-year outcomes (Fig. 6C). Notably, the prognostic performance of the Risk score surpassed that of preoperative PSA concentrations (Fig. 6D), indicating that this gene signature has good robustness and reproducibility across different cohorts.

Preoperative PSA level, clinical stage, and pathological stage are well-recognized clinical factors associated with prostate cancer prognosis. In this study, we further aimed to combine the molecular risk score with these macroscopic clinical features to jointly guide risk stratification and treatment decision-making. To identify prognosis-related clinical variables, we performed univariate Cox regression on common clinical characteristics and found that preoperative PSA concentration, clinical stage, pathological stage, and Gleason score had hazard ratios greater than 1 with *P* values less than 0.05 and could therefore be regarded as clinical risk indicators (Fig. 6E).

Based on these findings, we applied a LightGBM machine learning algorithm and incorporated the risk score together with PSA, clinical stage, pathological stage and Gleason score as input variables to train a “risk score–clinical feature” integrated prognostic model for prostate cancer. Feature importance analysis showed that the risk score contributed the most to the model (Fig. 6F), suggesting that this pathway related gene signature occupies a central position within the integrated model. Compared with any single clinical variable or the molecular index alone, the combined model showed superior accuracy in prognostic prediction, and its predictive performance was further confirmed in the TCGA validation cohort (Fig. 6G, H). These results indicate that a machine learning model that integrates pathway-based molecular features with clinical characteristics may provide a more refined and individualized prognostic tool for patients with prostate cancer. From a translational perspective, a molecular classification and risk score based on HAAO expression may be used to identify high-risk patients, optimize follow-up strategies, and assist treatment decisions. In addition, targeting HAAO and its related metabolic pathways, such as in combination with MVA pathway inhibition or AR pathway blockade, may offer new metabolic intervention strategies or combination treatment options for prostate cancer.

### HAAO^high^ prostate cancer displays synergistic vulnerability to combined inhibition of the MVA and AR pathways

**Fig. 7:**
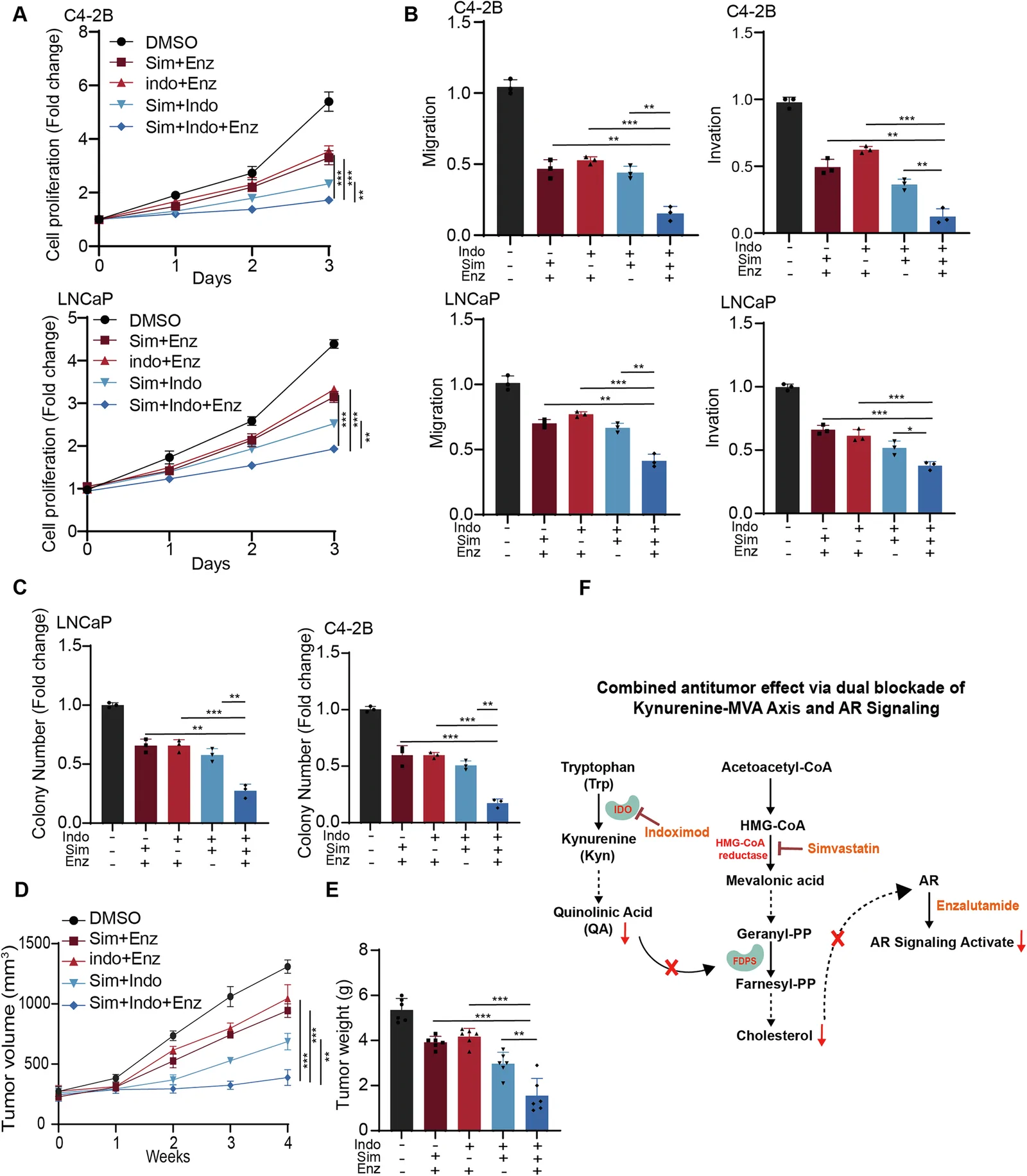
Combined inhibition of the MVA pathway and AR signaling reveals enhanced vulnerability in HAAO^high^ prostate cancer. **A** CCK-8 assay of LNCaP and C4-2B cells treated with DMSO, Sim plus Enz, Indo plus Enz, Sim plus Indo, or the triple combination of Sim, Indo, and Enz. Data are presented as mean ± SD. Statistical analysis was performed using one-way ANOVA followed by Dunnett’s multiple comparisons test. ∗∗*p* < 0.01, ∗∗∗*p* < 0.001. Sim: simvastatin, Indo: indoximod, Enz: enzalutamide. **B** Transwell assay showing migration and invasion ability of LNCaP and C4-2B cells treated with DMSO, Sim plus Enz, Indo plus Enz, Sim plus Indo, or the triple combination of Sim, Indo, and Enz. Data are presented as mean ± SD, and endpoint data were analyzed using one-way ANOVA followed by Dunnett’s test for multiple comparisons. ∗*p* < 0.05, ∗∗*p* < 0.01, ∗∗∗*p* < 0.001. Sim: simvastatin, Indo: indoximod, Enz: enzalutamide. **C** Colony formation assay of LNCaP and C4-2B cells treated with DMSO, Sim plus Enz, Indo plus Enz, Sim plus Indo, or the triple combination of Sim, Indo and Enz. Data are presented as mean ± SD, and endpoint data were analyzed using one-way ANOVA followed by Dunnett’s test for multiple comparisons. ∗∗*p* < 0.01, ∗∗∗*p* < 0.001. Sim: simvastatin, Indo: indoximod, Enz: enzalutamide. Tumor volumes (**D**) and endpoint weights (**E**) of LNCaP allografts in wild-type BALB/c mice treated with DMSO, Sim plus Enz, Indo plus Enz, Sim plus Indo, or the triple combination of Sim, Indo, and Enz. For (**D**), data are presented as mean ± SE, and endpoint data were analyzed using one-way ANOVA followed by Dunnett’s test for multiple comparisons. For (**E**), data are presented as mean ± SD, one-way ANOVA followed by Dunnett’s test for multiple comparisons. ∗∗*p* < 0.01, ∗∗∗*p* < 0.001. Sim: simvastatin, Indo: indoximod, Enz: enzalutamide. **F** Schematic showing how the tryptophan metabolite QA influences tumor cells to promote tumor progression. Solid arrows indicate direct biochemical reactions or metabolic conversions. Dashed arrows represent indirect regulatory effects or signaling pathways. Red cross symbols denote inhibition or blockade of the indicated processes.

Our previous results demonstrated that combined inhibition of the kynurenine pathway and the MVA pathway significantly suppressed the viability of prostate cancer cell lines, particularly in AR-positive cell lines (Fig. 4L). Given this enhanced sensitivity, we next investigated whether co-targeting AR signaling could further improve therapeutic efficacy. As shown in Fig. 7A–C, dual combinations treatment can inhibit cell proliferation, and the effects were strengthened by co-treatment of enzalutamide. To further validate these findings in vivo, we established C4-2B xenografts in nude mice. Consistent with the in vitro results, the triple combination induced significantly greater tumor growth inhibition compared with the dual combination treatment (Fig. 7D, E). Notably, C4-2B cells showed a stronger response to the combined treatment than LNCaP cells. This may reflect HAAO driven increases in MVA metabolic flux and maintenance of AR signaling in C4-2B, making these cells more dependent on this metabolic–hormonal axis for growth. This suggests that the expression level of HAAO may serve as a molecular marker to identify patients who are more likely to benefit from the combined treatment and supports the feasibility of HAAO as a potential clinical therapeutic target.

Taken together, these results indicate that there is functional cooperation among the kynurenine pathway, the MVA pathway, and AR signaling. Combined blockade of the kynurenine pathway, MVA pathway, and AR signaling can intervene at multiple levels of signal transduction and metabolic reprogramming and represents a promising metabolic targeting strategy for the treatment of prostate cancer (Fig. 7F).

## Discussion

Increasing evidence suggests that amino acids and their metabolites serve as important energy sources for tumor cells and participate in key metabolic pathways to provide the nutritional and signaling support required for rapid proliferation. Tryptophan metabolism has emerged as a critical metabolic axis that links metabolic reprogramming to immune regulation through the kynurenine pathway. Previous studies have shown that increased expression of IDO1 and TDO2 enhances the conversion of tryptophan to kynurenine, leading to activation of AhR signaling, suppression of effector T-cell activity, induction of Treg differentiation, and enhanced tumor immune tolerance [17]. Notably, this metabolic alteration also appears to be relevant in prostate cancer. Published human metabolic studies have reported enrichment of tryptophan metabolism along the kynurenine pathway in prostate cancer, including increased kynurenine and anthranilic acid in serum, as well as alterations in tryptophan and QA related metabolites in expressed prostatic secretion urine samples [14, 18]. In this study, we identified a subset of epithelial cells characterized by enhanced kynurenine pathway activity and unmasked a key communication axis linking tryptophan metabolism to lipid metabolism that drives prostate cancer progression. Aberrant activation of the kynurenine pathway, driven by HAAO, led to the accumulation of QA, which supports cholesterol-dependent AR signaling and tumor cell proliferation through FDPS stabilization. Knockdown of HAAO or blockade of QA influx using kynurenine-pathway inhibitors markedly suppressed the biological activity of prostate cancer cells, whereas combining QA inhibition with AR signaling blockade produced a more pronounced inhibitory effect on tumor progression.

Using single-cell transcriptomic analysis, we identified an epithelial subpopulation with markedly elevated tryptophan metabolic activity, in which the kynurenine pathway was selectively activated. Compared with bulk RNA-sequencing and tissue-level analyses, the single-cell approach enables resolution of metabolic heterogeneity at the cellular level and avoids signal averaging across mixed cell populations. Through single-cell profiling with pathway activity scoring and experimental validation, our analysis provides a more accurate assessment of kynurenine pathway dysregulation in prostate cancer and highlights the utility of single-cell approaches for dissecting metabolic states within heterogeneous tumors.

In this study, we further delineated the upstream regulatory mechanism underlying activation of the kynurenine pathway and identified HAAO as a key metabolic regulator that sustains kynurenine metabolism in prostate cancer. Enhanced HAAO activity leads to accumulation of QA within tumor cells. QA usually acts as an oxidizable substrate in the de novo NAD⁺ biosynthesis pathway, thereby providing metabolic support required for rapid cancer cell proliferation and biosynthesis [19]. Beyond its metabolic role, QA can also activate NMDA/PPARγ signaling in immune cells, contributing to the establishment of an immunosuppressive tumor microenvironment [20]. Importantly, our study reveals for the first time that QA functions as a structural modulator that directly binds to FDPS, a key enzyme in the MVA pathway, thereby stabilizing its conformation. This finding expands current understanding of metabolite–protein interactions and uncovers a previously unrecognized regulatory mode governing metabolic control in cancer cells. Moreover, this mechanism provides a new explanation for how tumor cells sustain AR signaling under androgen-deprived conditions, suggesting that the HAAO/QA–FDPS axis may contribute to the development of castration resistance (CRPC). These insights collectively highlight the biological and clinical significance of this metabolic axis in prostate cancer progression.

Emerging studies suggest that amino acid metabolic intermediates can directly bind to proteins and modulate their structure or activity. For example, lactate has been reported to modify proteins through covalent lysine lactylation, thereby directly linking cellular metabolic states to protein regulation and gene expression [21]. In line with this concept, our study demonstrates that QA interacts with FDPS through non-covalent binding and enhances its structural stability. Nevertheless, whether QA acts selectively on FDPS or exerts broader effects on additional protein targets remains unclear and will require systematic proteomic analyses and metabolite–protein interaction profiling. In addition, it is unknown whether the HAAO/QA–FDPS axis is universally present across different molecular subtypes of prostate cancer or restricted to specific genomic or metabolic contexts. Addressing this question will require validation in larger patient cohorts, integrated multi-omics datasets, and spatial profiling studies. Furthermore, although our study focuses primarily on tumor epithelial cells, it remains to be determined whether QA also regulates other components of the tumor microenvironment, such as immune cells or fibroblasts, and whether such effects influence tumor ecosystem dynamics. Finally, the present work is largely based on androgen-sensitive prostate cancer cell lines (LNCaP and C4-2B), and the activity and functional relevance of the HAAO/QA–FDPS axis in castration-resistant prostate cancer (CRPC) have not yet been explored. Consequently, its contribution to CRPC development remains uncertain and warrants investigation in therapy-resistant cell lines, organoid models, and in vivo CRPC systems. Future studies will aim to systematically map the QA-mediated protein interaction network and to determine whether the HAAO/QA–FDPS axis contributes to cellular adaptation following androgen-deprivation therapy.

In summary, QA derived from HAAO-high epithelial cells promotes FDPS stability and activates the mevalonate pathway to drive cholesterol-dependent AR signaling, thereby contributing to prostate cancer progression. For the first time, we identified lysine 257 as the QA binding site on FDPS, which is essential for its stability and function. Inhibition of QA influx by simvastatin, together with blockade of AR signaling, markedly suppresses tumor growth. Our study suggests that targeting the metabolic crosstalk among kynurenine metabolism, the MVA pathway, and AR signaling may offer a promising therapeutic strategy for precise metabolic intervention in castration-resistant prostate cancer.

## Materials and methods

### Single-cell RNA-seq data analysis

We collected publicly available prostate cancer scRNA-seq datasets consisting of 44 samples from 38 patients who had undergone or not undergone androgen deprivation therapy (Supplementary Table S1).

The single-cell RNA-seq matrix was analyzed using Seurat (version 5.0.2) [22]. Low-quality cells, defined as those with fewer than 200 or more than 6000 detected genes, or with more than 20% mitochondrial UMI counts, were excluded. After quality control, following the standard protocol of Seurat, the count data were normalized using the *NormalizeData* function, and the logarithm-transformed normalized matrix was used for downstream analyses. Highly variable genes (*n* = 2000) were identified using the “vst” method, followed by principal component analysis (PCA). Batch effects across datasets and samples were corrected using Harmony (version 1.2.1) [23]. A shared nearest neighbor (SNN) graph was constructed, and unsupervised clustering was performed with a resolution of 0.1. Cells were visualized using Uniform Manifold Approximation and Projection (UMAP).

### Broad cell type annotation

Based on canonical cell markers, the cells were finally classified into seven major subpopulations: B cells (CD79A, CD79B, CD19, MS4A1), Endothelial cells (PECAM1, VWF, PLVAP), Epithelial cells (EPCAM, KRT8, KRT18), Fibroblasts (COL1A1, DCN, COL1A2, COL3A1), Mast cells (TPAB1, TPSB2, KIT), Myeloid cells (CD68, CD163, CD14, C1QA, C1QB), and T/NK cells (CD2, CD3D, CD3E, PTPRC, KLRD1).

### Identifying malignant epithelial cells

To accurately distinguish tumor epithelial cells, we first used the *subset* function of Seurat to extract cells annotated as “Epithelial Cells”. Subsequently, copy number variation (CNV) in epithelial cells was inferred using CopyKAT (version 1.1.0) [24] with default parameters.

### cNMF analysis

Malignant epithelial cells were defined as patient-derived aneuploid epithelial cells identified by CopyKAT. Samples with fewer than 300 malignant epithelial cells were excluded. Raw count matrices were extracted from the RNA assay for each retained sample, and genes with zero total counts were removed before cNMF analysis. cNMF was performed on the merged AnnData object using the cNMF preprocessing workflow. Batch effects were corrected with Harmony using sample identity as the covariate, and 3000 highly variable genes were selected. Variance-normalized corrected expression matrices. cNMF was then run with component numbers ranging from 5 to 16, using 50 iterations and a random seed of 14. After combining replicate factorizations, the optimal rank was selected by k-selection analysis, and the final consensus solution was derived at *k* = 9 with a density threshold of 0.04.

### Scoring cells using gene expression signatures

Gene expression signatures related to tryptophan metabolism, including the kynurenine and 5-hydroxytryptamine (5-HT) pathways, were obtained from the KEGG database. All gene signatures used in this study are listed in Supplementary Table S2. Gene set variation analysis (GSVA, version 2.0.7) [25] was performed with default parameters to score pathway activity in malignant epithelial cells. For each malignant epithelial subset, mean GSVA scores were calculated across all cells. Differences in pathway activity between kynurenine and 5-HT pathways were assessed using the Wilcoxon rank-sum test.

### Hallmark pathway enrichment analysis

To identify biological pathways associated with differentially expressed genes (DEGs), gene set enrichment analysis (GSEA) was performed using the Hallmark gene set collection from the Molecular Signatures Database (MSigDB version 2023.1) [26].

### Bulk RNA-seq data analysis

Differentially expressed genes (DEGs) were identified using thresholds of |log₂ fold change| > 1 and adjusted *p* < 0.05. Functional enrichment analyses were performed using the clusterProfiler R package (version 4.14.3) [27] for Gene Ontology (GO) and KEGG pathways. GO terms and KEGG pathways with adjusted *p* < 0.05 and at least five overlapping genes were considered significant. Ingenuity Pathway Analysis (IPA, QIAGEN) was performed to identify enriched canonical pathways and upstream regulators associated with differentially expressed genes (DEGs). DEGs meeting the threshold of adjusted *p* < 0.05 were uploaded into IPA, and analyses were conducted using default settings. Canonical pathways and upstream regulators with an absolute *z*-score ≥ 2 and *p* < 0.05 were considered significant.

### Cell culture

Human prostate cancer cell lines LNCaP and C4-2B were obtained from the American Type Culture Collection (ATCC; Manassas, VA, USA) and cultured according to the supplier’s instructions. Cells were maintained in RPMI-1640 medium (Gibco) supplemented with 10% fetal bovine serum (FBS; Gibco) and 1% penicillin–streptomycin at 37 °C in a humidified incubator with 5% CO₂. Mycoplasma contamination was routinely tested by PCR, and cell line authentication was confirmed using short tandem repeat (STR) profiling. The cumulative culture length between thawing and experimental use was less than 15 passages.

### Western blotting

Western blotting analyses were carried out as described previously [28]. Antibody information is summarized in Supplementary Table S3. Full, uncropped Western blot images are provided in the Supplemental Material.

### RNA isolation and real-time qPCR analysis

Total RNA was extracted using TRIzol reagent (Vazyme) (Biotech, R401-01 reagent kit) and reverse transcribed using the ReverTra Ace qPCR RT kit (Toyobo, PCR-311) according to the manufacturer’s protocol. Real-time qPCR was performed using SYBR Green mix (Toyobo, QPK-201). The primer sequences used are listed in Supplementary Table S4.

### Transient transfection and viral transduction

Human HAAO plasmids were purchased from Sangon Biotech (Shanghai, China). All siRNAs were purchased from Genepharma (Guangzhou, China), and the sequences of siRNAs are listed in Supplementary Table S5. Lipofectamine 3000 (Invitrogen, Carlsbad, CA) was used for transfection following the manufacturer’s instructions. To avoid off-target effects, we transfected two siRNAs for better interference efficiency. Expression vectors for HAAO were purchased from LST Bio-tech Shandong Co., Ltd, and validated by immunoblotting. To acquire a stable cell line, single-cell clonal isolates were selected by 2 μg/ml puromycin in the culture media for 2 weeks.

### Cell proliferation, colony formation, migration, and invasion assays

Cell proliferation was determined by CCK-8 and EdU assays. Cells were seeded in 96-well plates and treated according to different experimental purposes. CCK-8 (Beyotime, Shanghai, China) was added at the indicated time points, and absorbance was measured. For the EdU assay, cells were seeded in 96-well plates. After 24 h, they were treated according to different experimental purposes, followed by incubation with 5-ethynyl-2′-deoxyuridine (EdU, Beyotime, Shanghai, China) for 2 h, and processed according to the manufacturer’s protocol. EdU-positive cell density was calculated with ImageJ software based on nuclear-specific staining (Hoechst 33342) compared with negative controls. For the colony formation assay, cells were seeded in 6-well plates (2000 cells/well). After 14 days, cells were fixed with 4% paraformaldehyde for 10 min and then stained with 0.5% crystal violet for 20 min. Colonies were counted using ImageJ software. Cell migration and invasion were assessed using Transwell chambers (Corning, NY, USA) with or without Matrigel coating.

### Tumor models

Male nude mice aged 4–6 weeks were purchased from Huafukang Biotechnology (Beijing, China) and maintained under specific pathogen-free (SPF) conditions. All animal experiments were conducted in accordance with institutional guidelines for the care and use of laboratory animals.

For xenograft establishment, 5 × 10⁶ LNCaP or C4-2B cells stably overexpressing HAAO, along with their corresponding control cells, were suspended in 100 μL of PBS containing 50% Matrigel and subcutaneously injected into the flanks of the mice. Tumor volume was measured every 4 days and calculated as Volume = 0.5 × length × width². Mice were randomly assigned (*n* = 6 per group) and treated once every 2 days with either QA (100 mg/kg, i.v.) or vehicle control (DMSO). For combination treatment, mice were randomly divided into groups (*n* = 6 per group) and administered indoximod (10 mg/kg, i.p.), simvastatin (10 mg/kg, i.p.), or vehicle (DMSO, i.p.) once every 2 days.

### Flow cytometry

LNCaP and C4-2B cells were treated with Tm/Tg to induce apoptosis and subsequently cultured with or without QA. Cells were harvested, stained with Annexin V–FITC and propidium iodide (PI) following the manufacturer’s protocol, and analyzed by flow cytometry. The percentages of apoptotic cells were quantified and compared across groups.

### ELISA of QA secretion

C4-2B and LNCaP cells were seeded into 6-well plates and treated according to different experimental purposes. After 36 h, cells were cultured in RPMI-1640 supplemented with 2% FBS for the indicated time, and the conditioned medium was subsequently collected. QA secreted into the medium was detected using an ELISA kit (Shbaiyibo, Shanghai, China).

### Drug affinity responsive target stability (DARTS)

The DARTS assay was performed as previously described [29]. Briefly, different concentrations of QA were added to an equal volume of LNCaP cell lysate and incubated at room temperature for 1 h. Then, the samples were digested with pronase at the specified ratios for 30 min at room temperature. After digestion, a loading buffer was added, and the samples were boiled and analyzed via Western blotting.

### Cellular thermal shift assay (CETSA)

Cellular thermal shift assay was performed as previously described [30]. In brief, LNCaP cells treated with DMSO or QA (100 µM) for 2 h. Cells were collected and resuspended in TBS. Multiple aliquots of cells were heated at 45, 50, 55, 60, and 65 °C for 3 min. Cells were lysed, precipitates were removed, and the FDPS in the soluble fraction was quantified by Western blot analyses.

### Metabolite extraction and quantification

Cellular cholesterol and geranylgeranyl pyrophosphate (GGPP) levels were extracted and quantified using established protocols.

### Molecular docking

The human FDPS structure was retrieved from the RCSB Protein Data Bank (https://www.rcsb.org) under PDB ID 1ZW5. Molecular docking was subsequently carried out with the Yinfotek online server (https://www.yinfotek.com).

### Risk score construction

To construct a pathway based prognostic signature, genes from the kynurenine pathway, the mevalonate (MVA) pathway, and the AR signaling pathway were curated from KEGG. A total of 142 pathway-related genes were included for initial screening. Death, biochemical recurrence (BCR), and lymph node metastasis were considered poor prognostic events.

Thirteen genes that consistently showed significant hazard ratios were selected for model construction and are listed in Supplementary Table S6. Risk score was calculated using the Cox regression coefficients and gene expression levels:

$${\rm{Risk\; Score}}=\mathop{\sum }\limits_{i=1}^{n}(\beta i\times {Expri})$$

### Integrated prognostic model development

A binary classification model was developed using the Light Gradient Boosting Machine (LightGBM). LightGBM is an efficient tree-based gradient boosting framework that iteratively fits decision trees to the residuals of previous models. To improve computational efficiency on high-dimensional data, LightGBM incorporates Gradient-based One-Side Sampling (GOSS) to reduce the number of training instances and Exclusive Feature Bundling (EFB) to compress mutually exclusive features. The model employs a leaf-wise tree growth strategy with depth constraints, enabling more effective reduction of the loss compared to traditional level-wise growth.

For this classification task, the objective function was set to the logistic loss. The dataset was randomly split into a training set (80%) and an independent test set (20%). Model development was conducted on the training set using 5-fold cross-validation to select optimal hyperparameters and assess model robustness. The final model was evaluated on the held-out test set to ensure unbiased estimation of generalization performance.

To interpret model behavior, feature importance was quantified using gain importance, which measures the total reduction in loss contributed by each feature across all splits of all trees. Reported feature importance values represent the average results across the five folds.

### Statistical analysis

Statistical analyses in this study were carried out using GraphPad Prism 8 (RRID:SCR_002798) and SPSS 22.0 (IBM Corporation) (RRID:SCR_002865). All experiments in vitro were performed in biological triplicate. The two-tailed unpaired t-test was used to calculate statistical significance between the two groups. Survival information was verified by Kaplan-Meier analysis and compared using the log-rank test. In the xenograft studies, tumor sizes were served as the primary response measure when the mice were sacrificed. The tumor growth was analyzed by ANOVA. *P* values considered to be significant as follows: *p < 0.05; **p < 0.01; ***p < 0.001 and ****p < 0.0001.

### Ethics approval and consent to participate

No human participants or human tissue samples were involved in this study. The studies involving animals (D20230117017) were reviewed and approved by the Ethics Committee of Shandong First Medical University. All methods were performed in accordance with the relevant guidelines and regulations.

## Supplementary information

Supplementary materials for HAAO-derived quinolinic acid fuels FDPS‑dependent AR signaling and sensitizes prostate cancer to combination therapy

Full, uncropped Western blot images

## Data Availability

All the scRNA-seq data used in this work are publicly available. Datasets retrieved from the Gene Expression Omnibus can be downloaded using the following accession numbers: GSE137829 [31], GSE141445 [32], GSE157703 [33], GSE193337 [34], GSE264573 [35], and GSE176031 [36]. The remaining datasets were downloaded directly from links provided in their corresponding publications: Wang Z et al. [37]. All data and materials during the current study are available from the corresponding author upon reasonable request.

## Code availability

The analysis in this study was performed using standard and publicly available R packages as detailed in the “Methods” section. No custom codes were developed for any analyses in this study. Therefore, no additional code is available for public sharing.
