# Supplementary material for: HAAO‑derived quinolinic acid fuels FDPS‑dependent AR signaling and sensitizes prostate cancer to combination therapy
Source: Cell Death Discov. 2026 Jun 16;12:344. doi: 10.1038/s41420-026-03203-x (PMC13503666; doi:10.1038/s41420-026-03203-x)

Full unedited blot/gel for Figure 4C

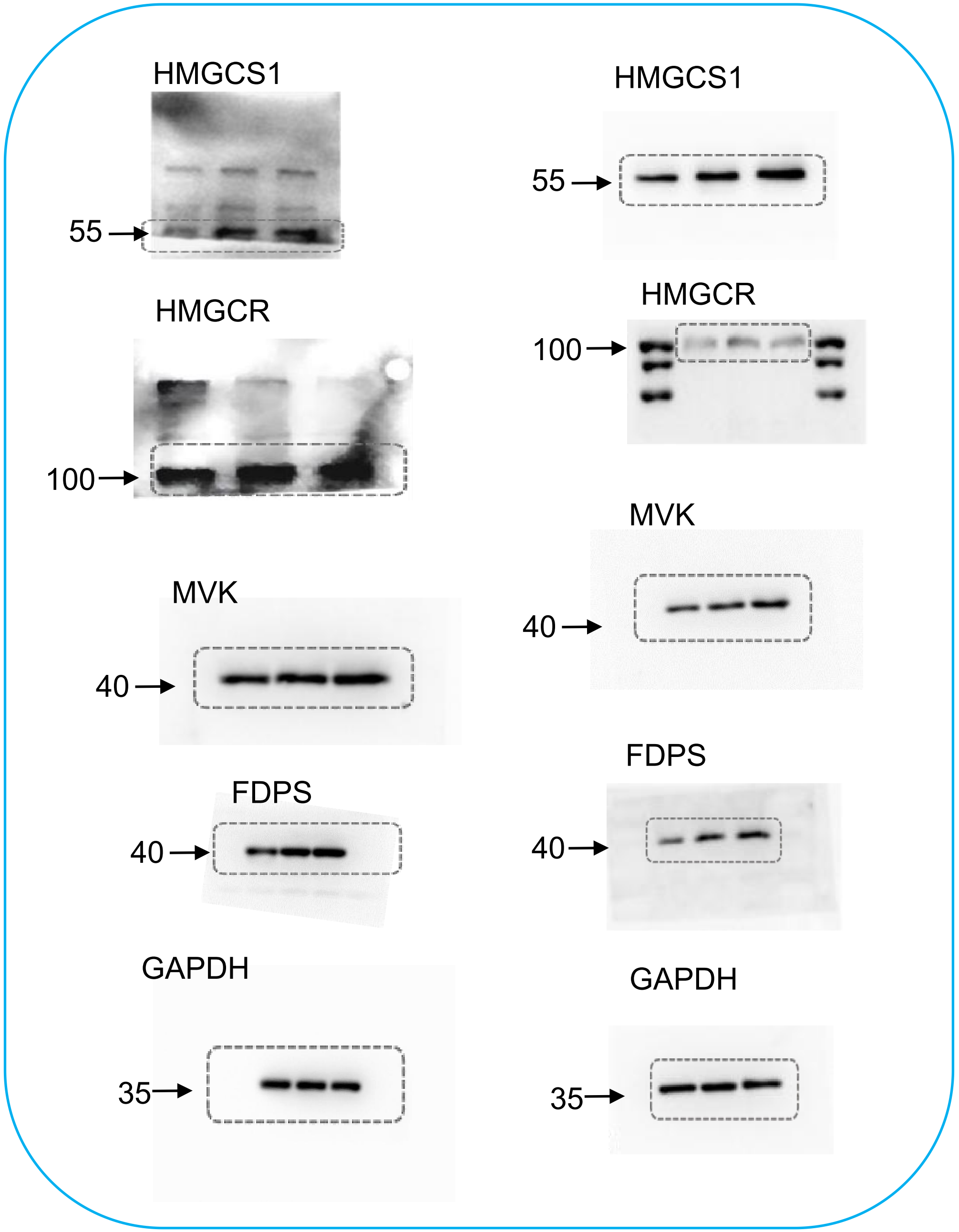

Full unedited blot/gel for Figure 4E left

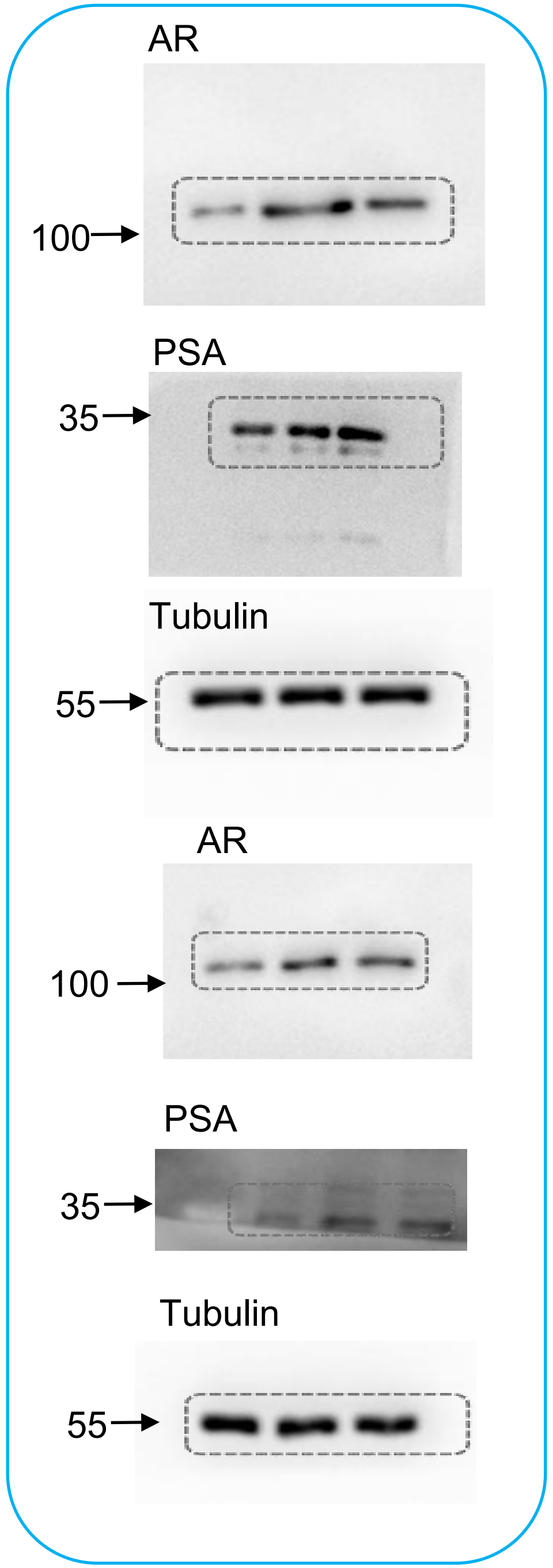

Full unedited blot/gel for Figure 4E right

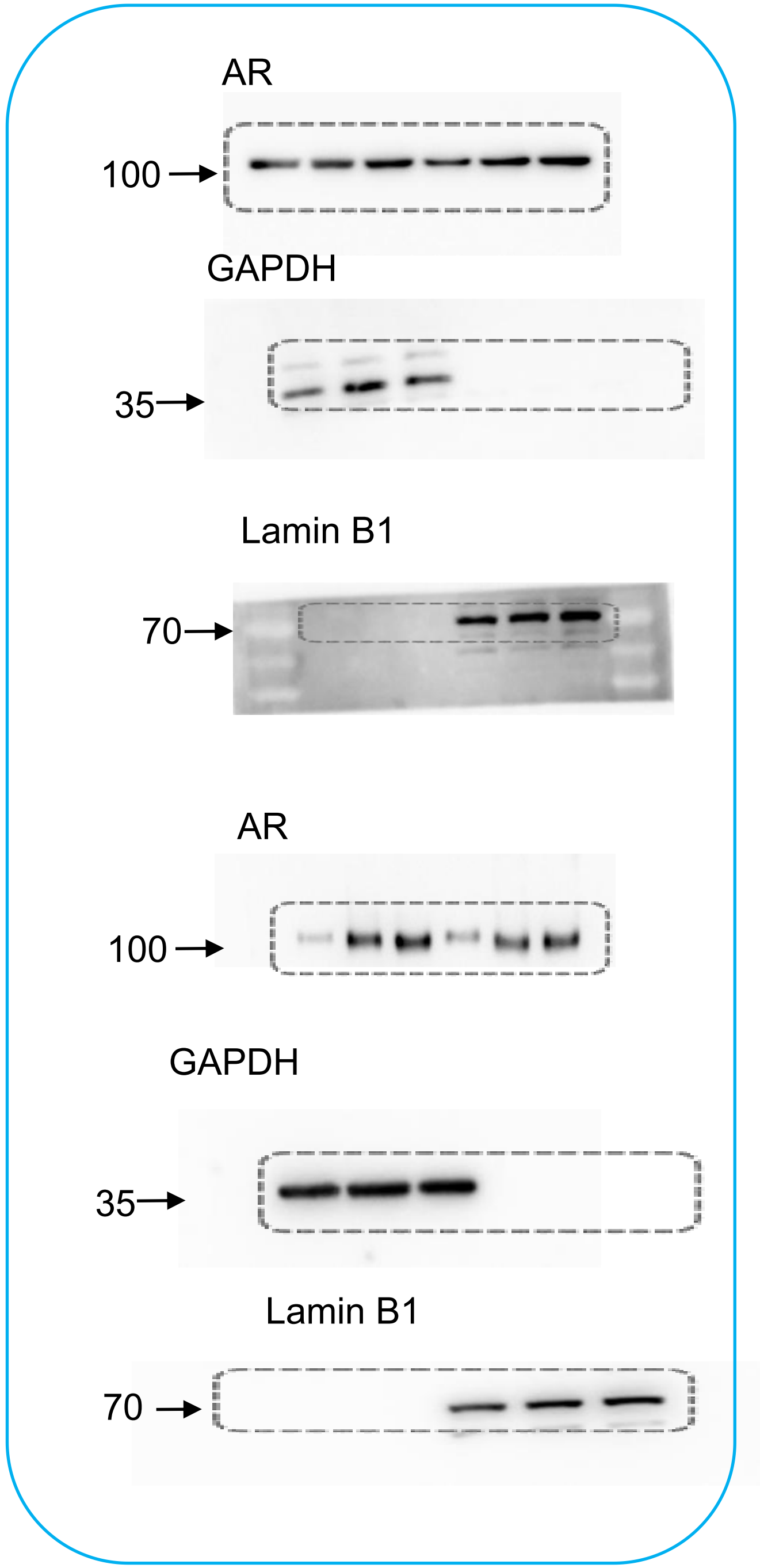

Full unedited blot/gel for Figure 4J

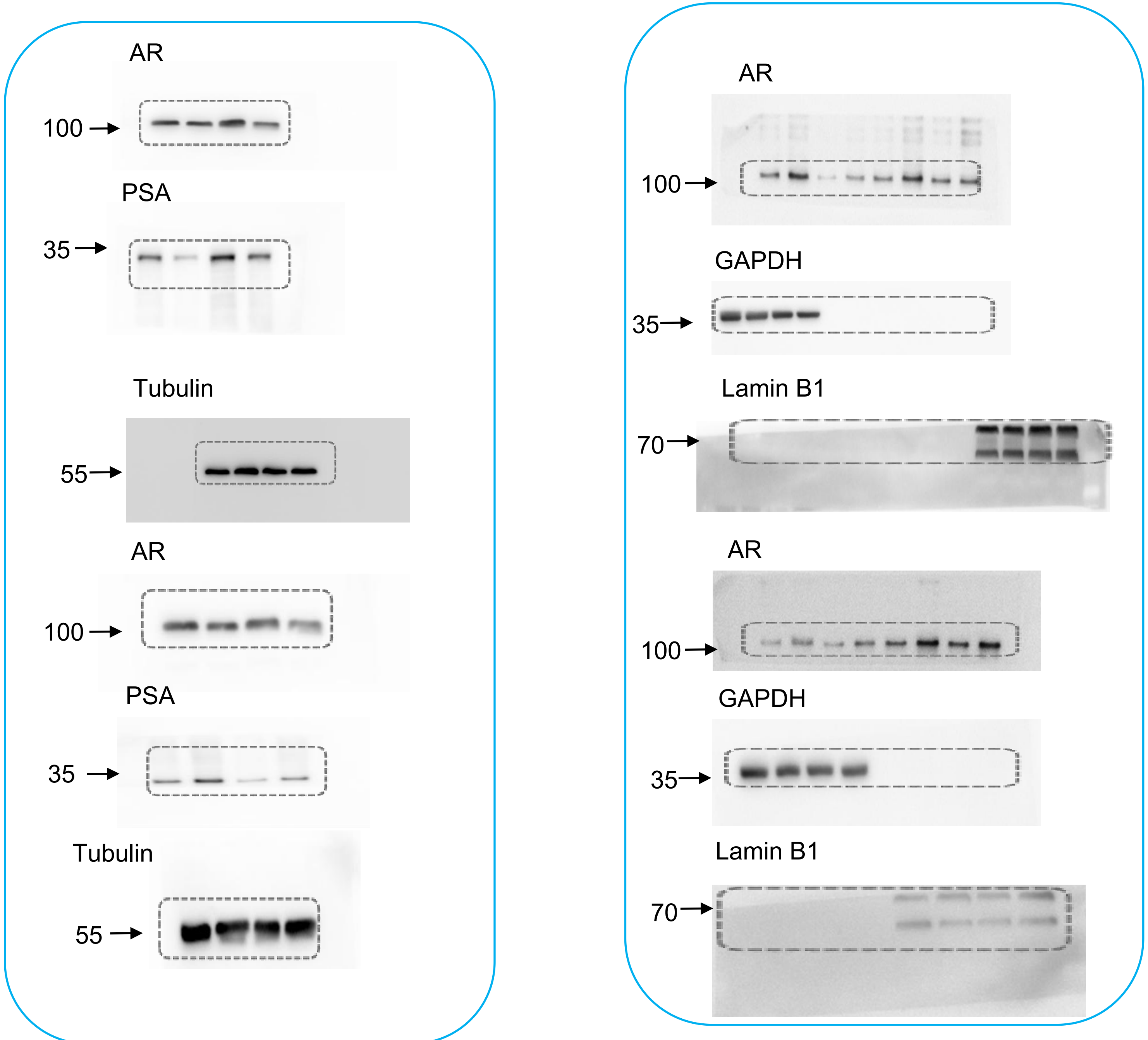

Full unedited blot/gel for Figure 5B

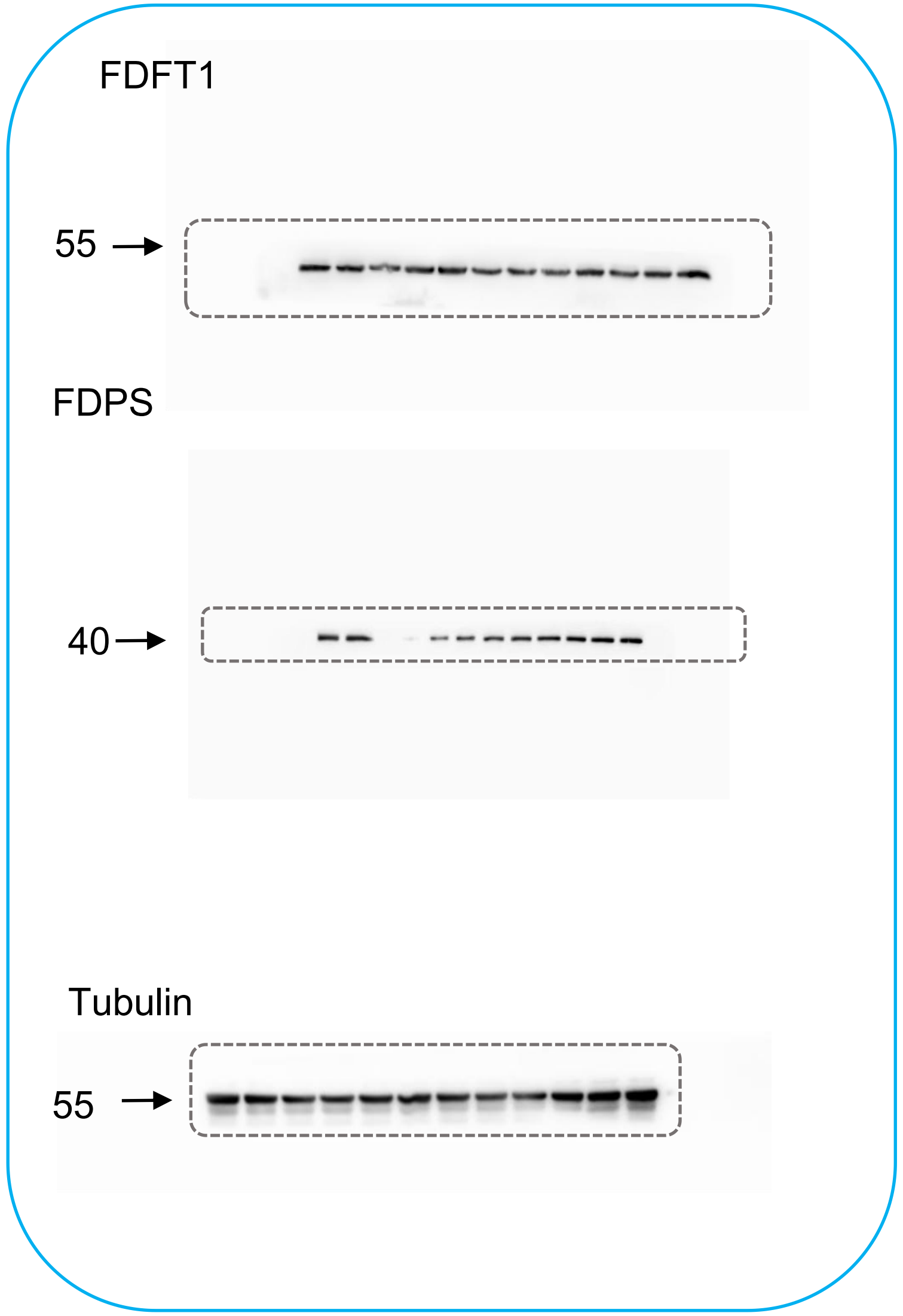

Full unedited blot/gel for Figure 5C

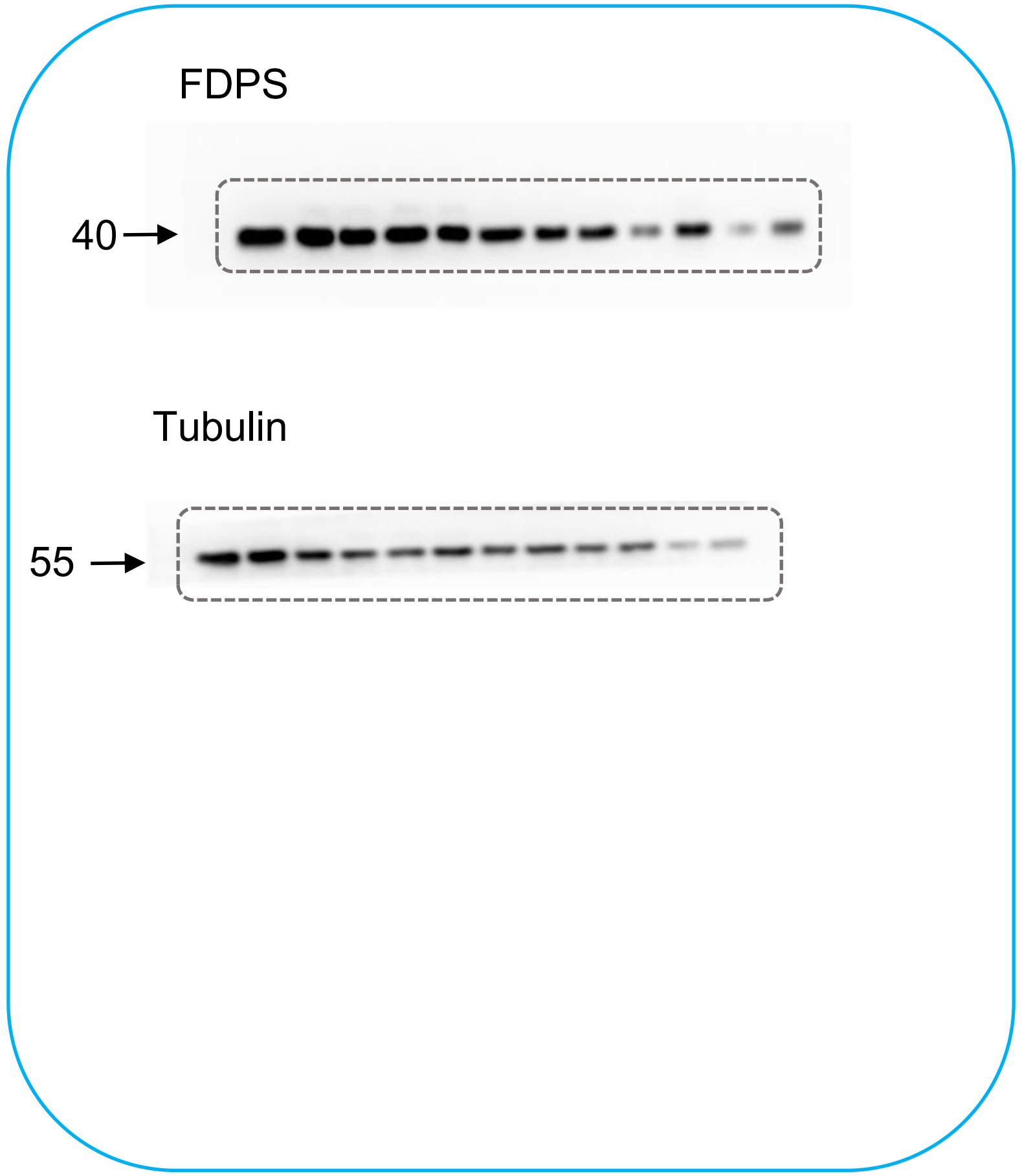

Full unedited blot/gel for Figure 5D

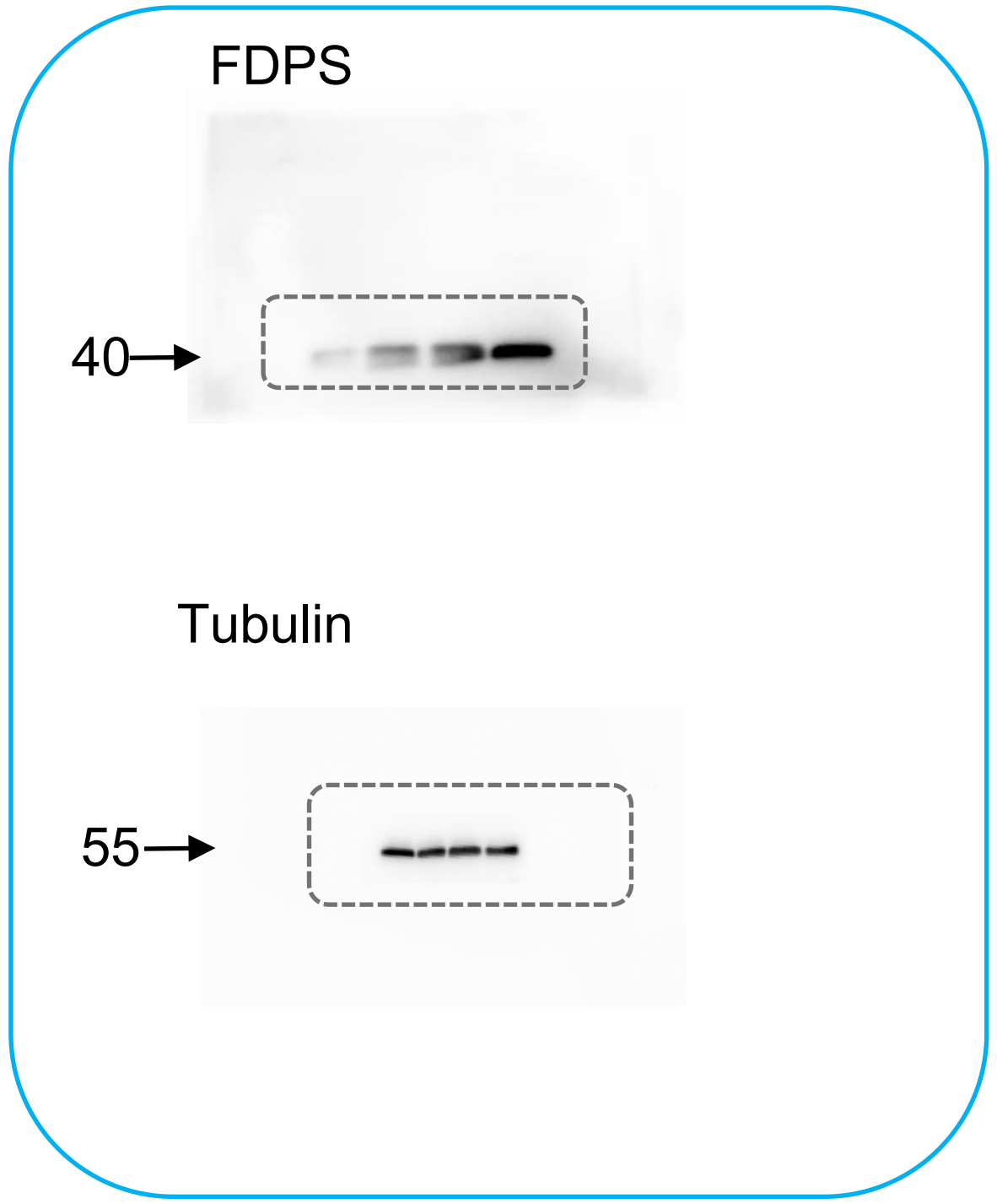

Full unedited blot/gel for Figure 5F

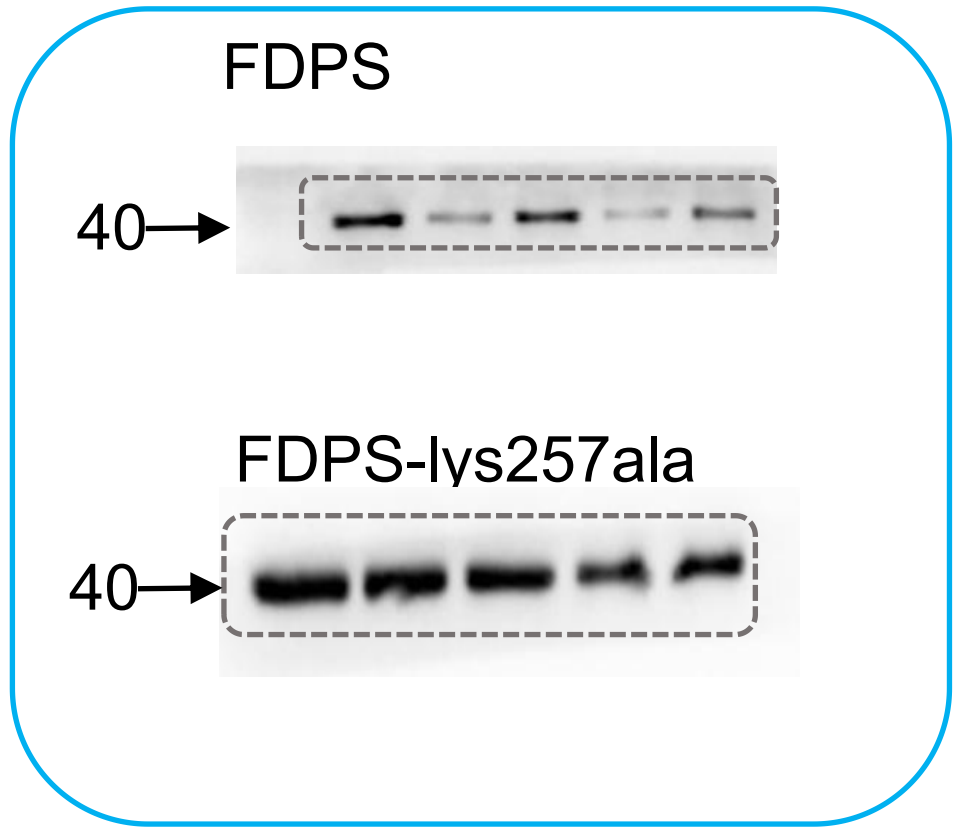

Full unedited blot/gel for Figure 5I left

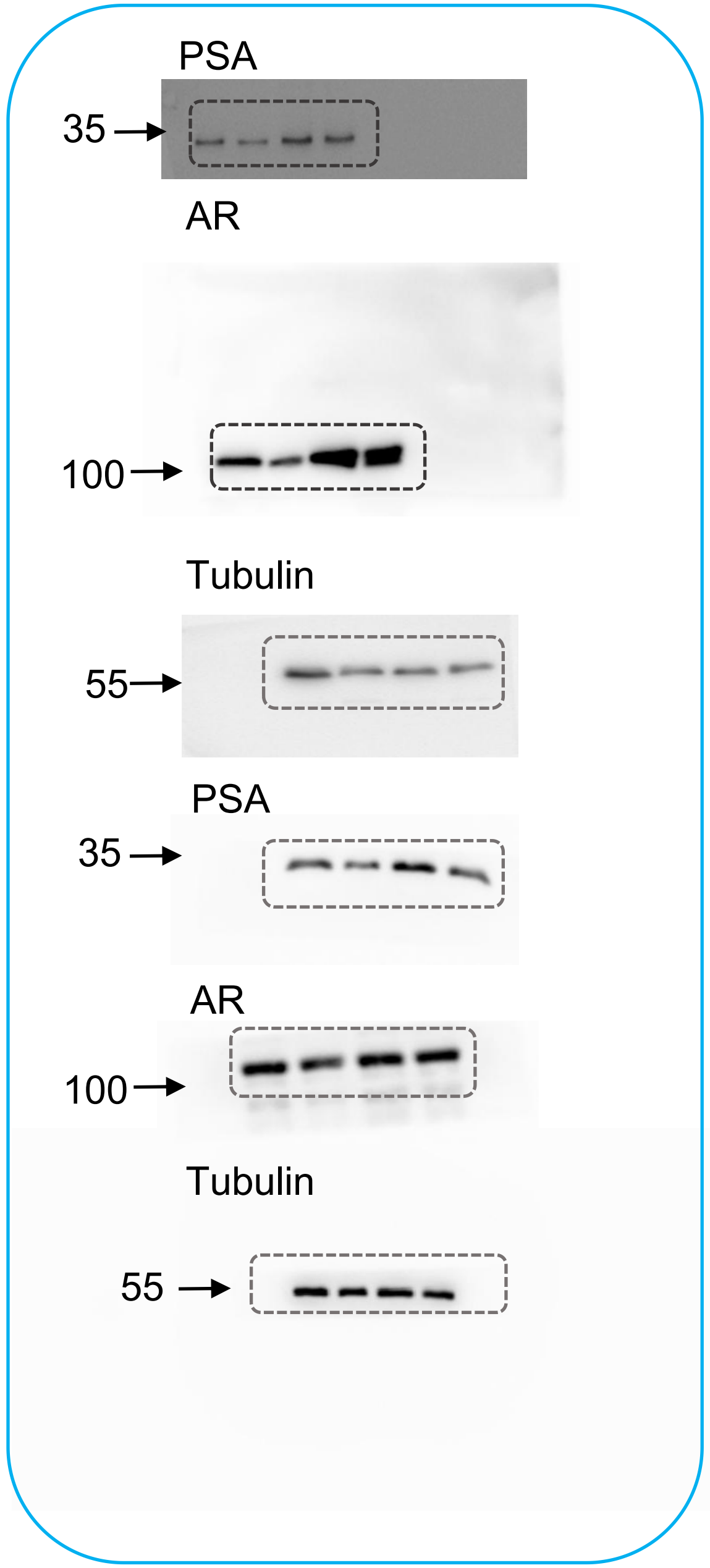

Full unedited blot/gel for Figure 5I right

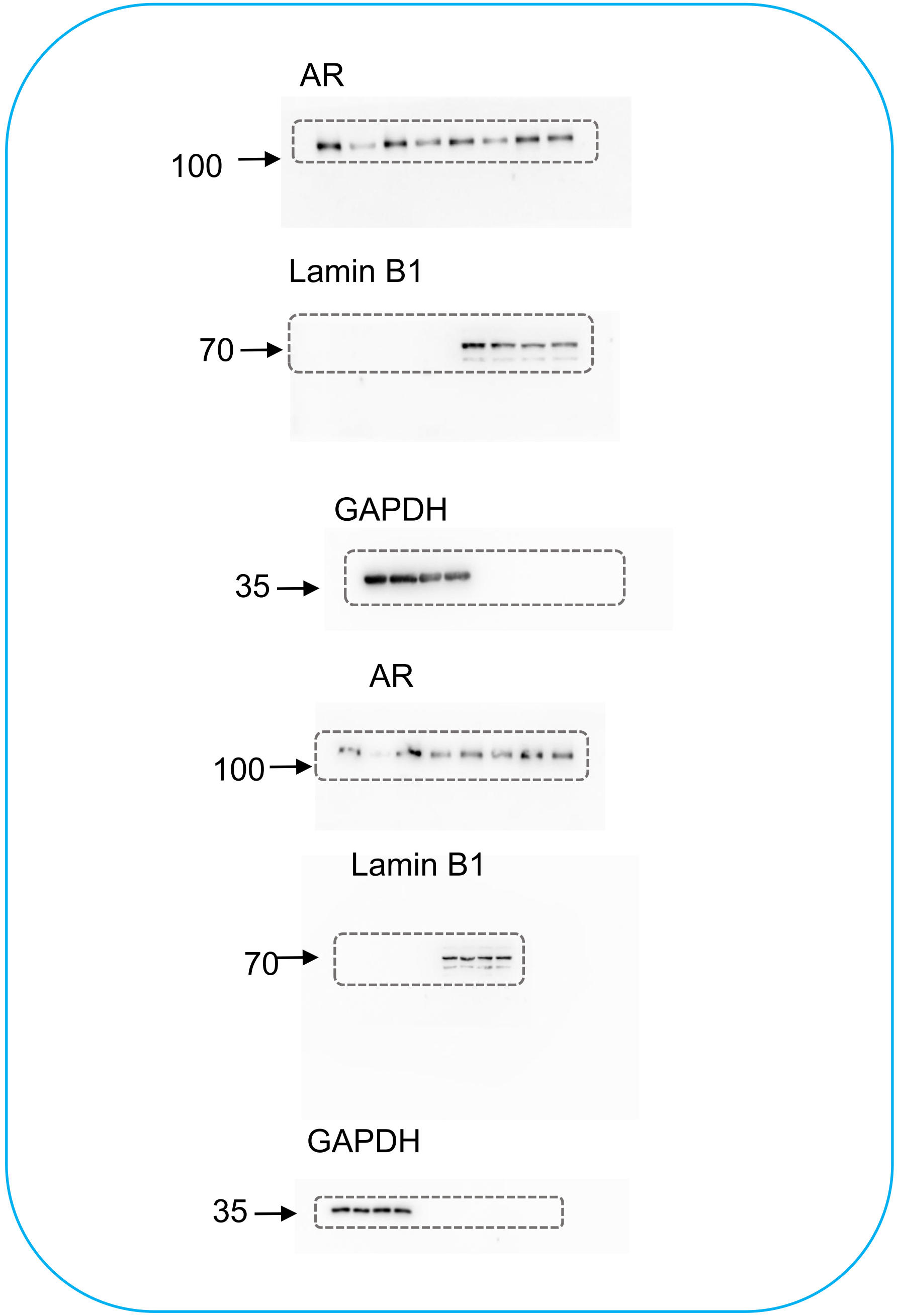

Full unedited blot/gel for Figure S2

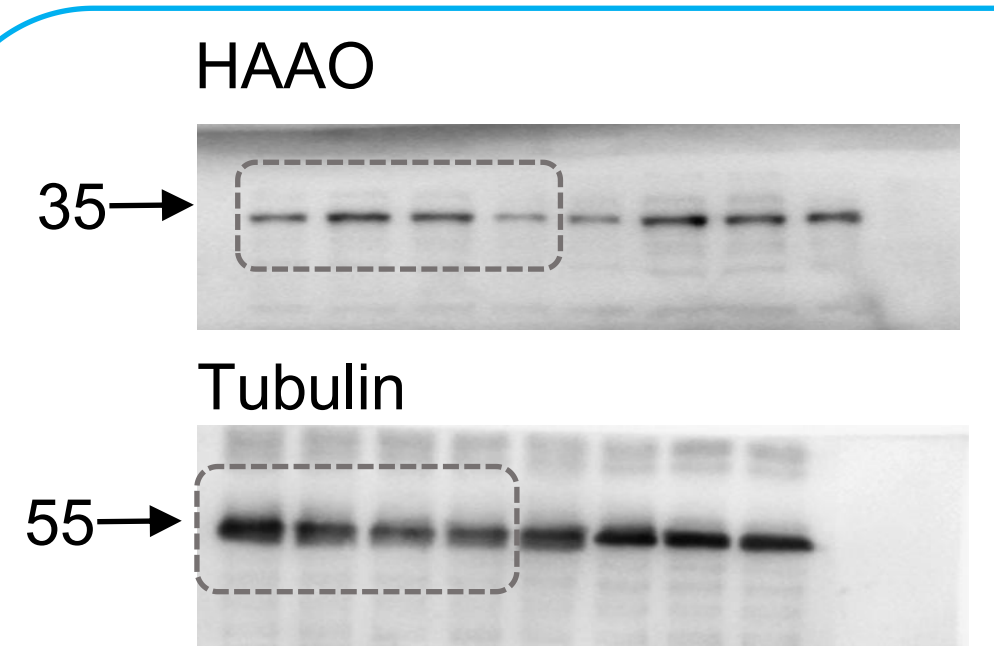

Full unedited blot/gel for Figure S4

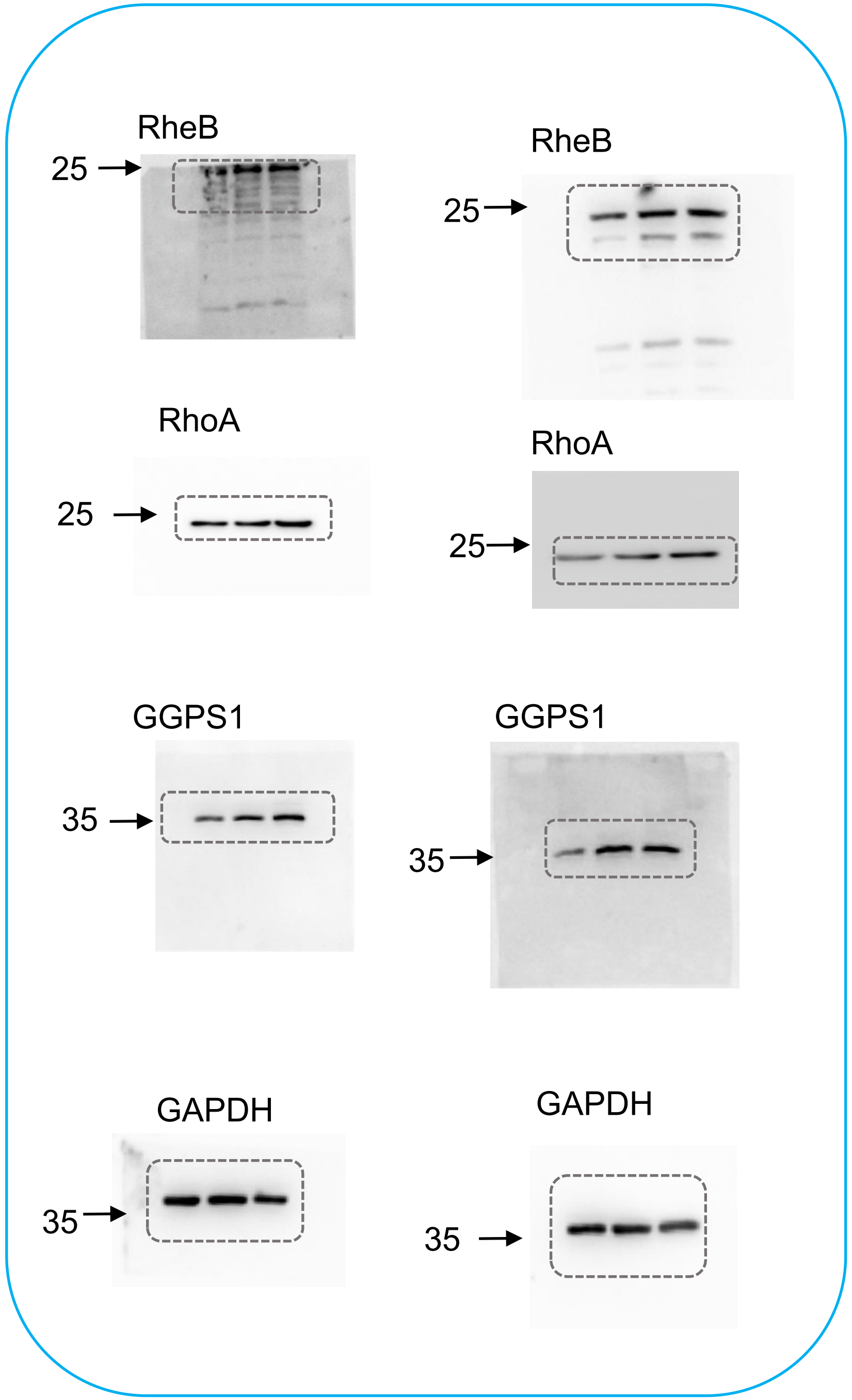

Supplement: Supplementary file 1 [file 41420_2026_3203_MOESM2_ESM.pdf]
