# Supplementary material for: HAAO‑derived quinolinic acid fuels FDPS‑dependent AR signaling and sensitizes prostate cancer to combination therapy
Source: Cell Death Discov. 2026 Jun 16;12:344. doi: 10.1038/s41420-026-03203-x (PMC13503666; doi:10.1038/s41420-026-03203-x)
Supplement: Supplementary file 2 [file 41420_2026_3203_MOESM1_ESM.docx]

Supplementary materials for

**HAAO-derived quinolinic acid fuels FDPS‑dependent AR signaling and sensitizes prostate cancer to combination therapy**

Hongchang Zhang *et al*.

*Correspondence:

**CONTENTS**

**Figure. S1-S6**

**Tables. S1 to S6**

**Figure S1**

**Figure S1. Single-cell transcriptomic landscape and kynurenine pathway activity across malignant epithelial cells.** **(A)** Integrated analysis of 123,986 cells from 7 published human scRNA-seq datasets. **(B)** UMAP plot showing the aneuploid, diploid and not defined epithelial cells in = prostate cancer tissues. **(C)** Box plot showing the GSVA scores of the kynurenine and 5-HT pathways in malignant cells. Wilcoxon rank-sum test used for comparison, ***p < 0.001. **(D)** Volcano plot showing differentially expressed genes in cluster C5 compared with other epithelial clusters. **(E)** KEGG analysis showing the top five enriched pathways for each malignant epithelial cluster. **(F)** Box plots showing stemness and EMT scores in cluster C5 compared with other epithelial subpopulations. **(G)** Stacked bar plot showing the distribution proportions of malignant epithelial clusters across various datasets. **(H)** Bubble plot showing the expression levels of kynurenine pathway genes in the C5 cluster.

**Figure S2**

**Figure S2. Validation of HAAO overexpression and knockdown at the protein and mRNA levels. (A)** Relative mRNA levels of HAAO in prostate cancer cells**. (B)** Western blot analysis of HAAO level in HAAO-OE LNCaP and siHAAO C4-2B. **(C)** The relative HAAO mRNA level in siHAAO C4-2B cells. Data are presented as mean ± SD. Unpaired two-tailed Student’s t-test used for comparison. ∗∗∗p < 0.001. **(D)** The relative HAAO mRNA level in HAAO-OE LNCaP cells. Data are presented as mean ± SD. Unpaired two-tailed Student’s t-test used for comparison. ∗∗∗p < 0.001.

**Figure S3**

(A) Box plot showing QPRT expression in primary vs normal tissue of prostate cancer patients across different datasets. Wilcoxon rank-sum test used for comparison, ***p < 0.001, ****p < 0.0001. **(B)** Bar plot showing the OD values (490 nm) of DU145,PC3,LNCaP and C4-2B cells treated with different QA concentrations. **(C)** CCK-8 assay of DU145 and PC3 cells treated with different QA concentrations. Data are presented as the mean ± SD. ∗∗p < 0.01. **(D)** Flow cytometry analysis showing the apoptosis levels of LNCaP and C4-2B cells after Tm/Tg induced apoptosis, with or without QA treatment. Data are presented as mean ± SD, and endpoint data were analyzed using one-way ANOVA followed by Dunnett’s test for multiple comparisons. ∗∗p < 0.01, ∗∗∗p < 0.001. **(E)** ELISA analysis of secreted QA levels in C4-2B and LNCaP cells. QA concentrations are presented as ng/mL. Data are presented as the mean ± SD.∗p < 0.05, ∗∗p < 0.01.

**Figure S4**

**Figure S4. QA modulates the MVA pathway in prostate cancer cells. (A)** Western blot analysis showing the expression of MVA pathway proteins in LNCaP and C4-2B cells treated with different QA concentrations. **(B)** GGPP levels of LNCaP and C4-2B cells treated with QA. **(C)** Schematic diagram showing the crosstalk between the kynurenine pathway and the mevalonate (MVA) pathway**. (D)** Bar chart show results from LNCaP and C42B cells treated under three conditions: (1) no QA or PHT; (2) PHT only; and (3) QA + PHT with simultaneous measurement of cholesterol levels. Data are presented as the mean ± SD.∗∗p < 0.01, ∗∗∗p < 0.001.

**Figure S5**

**Figure S5. QA associated proteomic and structural analyses. (A)** LNCaP cells were treated with 300 µM quinolinic acid (QA), followed by protease digestion of the cell lysates and Coomassie Brilliant Blue staining. **(B)** IPA analysis showing the top canonical pathways in LNCaP cells treated with QA. **(C)** IPA analysis showing the top canonical transcription factor in LNCaP cells treated with QA. **(D)** Molecular docking model showing the relative binding affinities of quinolinic acid at different sites.

**Figure S6**

**Figure S6. Kaplan–Meier survival analysis of genes stratified by high and low expression. (A)** Kaplan-Meier survival of high and low expression groups of survival-related genes.

**Table S1. Single cell RNAseq datastes**

| **Dataset** | **Patient ID** | **SampleID** | **Stage（TNM）** | **PSA** | **Gleason score** | **Site of Biopsy** |
| --- | --- | --- | --- | --- | --- | --- |
| **GSE137829** | **P1** | **GSM4089151-P1** | **cT2cN0M0** | **8.88** | **4+5=9** | **Prostate** |
|  | **P2** | **GSM4089152-P2** | **cT3bN1M0** | **56.53** | **7(4+3)** | **Prostate** |
|  | **P3** | **GSM4089153-P3** | **cT2cN0M0** | **55.16** | **8(4+4)** | **Prostate** |
|  | **P4** | **GSM4089154-P4** | **cT4N0M0** | **149** | **7(4+3)** | **Prostate** |
|  | **P5** | **GSM4089155-P5** | **cT3bN1M1a** | **15.6** | **8(4+4)** | **Prostate** |
|  | **P6** | **GSM4089156-P6** | **cT4N1M1** | **72.72** | **Small-cell NEPC** | **Prostate** |
| **GSE141445** | **P7** | **GSM4203181-1** | **cT4N1M0** | **79.44** | **9(4+5)** | **Prostate** |
|  | **P8** | **GSM4203181-2** | **cT4N0M0** | **18.51** | **10(5+5)** | **Prostate** |
|  | **P9** | **GSM4203181-3** | **cT3aN0M0** | **7.05** | **9(4+5)** | **Prostate** |
|  | **P10** | **GSM4203181-4** | **cT3aN0M0** | **22.57** | **9(4+5)** | **Prostate** |
|  | **P11** | **GSM4203181-5** | **cT2cN0M0** | **10.5** | **7(4+3)** | **Prostate** |
|  | **P12** | **GSM4203181-6** | **cT2cN1M0** | **336** | **7(4+3)** | **Prostate** |
|  | **P13** | **GSM4203181-7** | **cT2cN1M1b** | **16** | **9(4+5)** | **Prostate** |
|  | **P14** | **GSM4203181-8** | **cT2cN1M1b** | **16** | **9(4+5)** | **Prostate** |
|  | **P15** | **GSM4203181-9** | **cT2cN0M0** | **20** | **9(4+5)** | **Prostate** |
|  | **P16** | **GSM4203181-10** | **cT2cN0M0** | **81.09** | **7 (3 + 4)** | **Prostate** |
|  | **P17** | **GSM4203181-11** | **cT3aN0M1b** | **19** | **9(4+5)** | **Prostate** |
|  | **P18** | **GSM4203181-12** | **cT3aN0M0** | **51** | **7(4+3)** | **Prostate** |
|  | **P19** | **GSM4203181-13** | **cT2bN0M1a** | **14** | **9(4+5)** | **Lymph node** |
| **GSE157703** | **P20** | **GSM4773521_PCa1** | **pT2N0** | **NA** | **6 (3 + 3)** | **Prostate** |
|  | **P21** | **GSM4773522_PCa2** | **pT3aN1** | **NA** | **7 (3 + 4)** | **Prostate** |
| **GSE193337** | **P22** | **GSM5793828_P1t** | **pT2cN0** | **20** | **NA** | **Prostate** |
|  | **P23** | **GSM5793829_P2t** | **pT2aN0** | **2.4** | **NA** | **Prostate** |
|  | **P24** | **GSM5793831_P3t** | **pT3aN0** | **14** | **NA** | **Prostate** |
|  | **P25** | **GSM5793832_P4t** | **pT3bN0** | **2.32** | **NA** | **Prostate** |
| **GSE264573** | **P26** | **GSM8222914_HMP22** | **NA** | **NA** | **NA** | **Lymph node** |
|  | **P27** | **GSM8222915_HMP23A** | **NA** | **NA** | **NA** | **Prostate** |
|  | **P28** | **GSM8222916_HMP23B** | **NA** | **NA** | **NA** | **Prostate** |
|  | **P29** | **GSM8222917_HMP24** | **NA** | **NA** | **NA** | **Pelvic Mass** |
| **PMID35789834** | **P30** | **PMID35789834-P1** | **cT3bN1M1b** | **8.63** | **9(4+5)** | **Lymph node** |
|  | **P31** | **PMID35789834-P2** | **cT1cNxMx** | **6.39** | **9(4+5)** | **Prostate** |
|  | **P32** | **PMID35789834-P3** | **cT3bN1M0** | **71.64** | **9(4+5)** | **Lymph node** |
|  | **P33** | **PMID35789834-P6** | **cT3bN1M1b** | **147.71** | **9(4+5)** | **Prostate** |
| **GSE176031** | **P34** | **GSM5353232_PA_PR5249_T1_S3** | **cT2b** | **5.2** | **7 (3 + 4)** | **left lateral gland** |
|  |  | **GSM5353233_PA_PR5249_T2_S4** |  |  |  |  |
|  | **P35** | **GSM5353236_PA_PR5251_T1_S7** | **cT2c** | **7.7** | **7 (3 + 4)** | **left mid** |
|  |  | **GSM5353237_PA_PR5251_T2_S8** |  |  |  |  |
|  | **P36** | **GSM5353240_PA_PR5254_T1_S15** | **cT2a** | **7.8** | **7(4+3)** | **left mid** |
|  | **P37** | **GSM5353243_PA_PR5261_T1_S23** | **cT2a** | **3.89** | **7 (3 + 4)** | **central mid towards apex** |
|  |  | **GSM5353244_PA_PR5261_T1_S24** |  |  |  |  |
|  | **P38** | **GSM5353245_PA_PR5269_1_S25** | **cT1c** | **4.1** | **7 (3 + 4)** | **NA** |
|  |  | **GSM5353246_PA_PR5269_2_S26** |  |  |  |  |
|  |  | **GSM5353247_PA_PR5269_3_S27** |  |  |  |  |
|  |  | **GSM5353248_PA_PR5269_4_S28** |  |  |  |  |

**Table S2. Gene expression signatures related to tryptophan metabolism**

| Kynurenine pathway | 5-hydroxytryptamine (5-HT) pathways |
| --- | --- |
| AFMID | TPH1 |
| HAAO | TPH2 |
| IDO1 | AADC |
| IDO2 | MAO |
| KMO | AANAT |
| KYNU | ASMT |

**Table S3. Antibodies**

| Antibody Target | Source | Application |
| --- | --- | --- |
| GAPDH | Selleckchem;Catalog No.: F0003 | WB |
| HMGCS1 | ABclonal;Catalog No.:A3916 | WB |
| HMGCR | ABclonal;Catalog No.:A1633 | WB |
| MVK | ABclonal;Catalog No.:A20906 | WB |
| FDPS | ABclonal;Catalog No.:A23475 | WB |
| RheB | ABclonal;Catalog No.:A1165 | WB |
| RhoA | ABclonal;Catalog No.:A0272 | WB |
| GGPS1 | ABclonal;Catalog No.:A16233 | WB |
| Rap1A | Thermo Fisher;Catalog No.:MA5-17164 | WB |
| Tubulin | Thermo Fisher;Catalog No.:MA5-16308 | WB |
| AR | Thermo Fisher;Catalog No.:MA5-13426 | WB |
| PSA | Thermo Fisher;Catalog No.:MA1-10447 | WB |
| Lamin B1 | Thermo Fisher;Catalog No.:702972 | WB |
| FDFT1 | Thermo Fisher;Catalog No.:MA5-44872 | WB |

**Table S4. Primers**

| Gene Name | Sequence (5´to 3´) |
| --- | --- |
| GAPDH | F: CTT GTG TTC GCT CCC AGG CAT A |
|  | R: GGC TGT TGT CAT ACT TCT CAT GG |
| HAAO | F: CCT GAG ACA GAA TG TGG ACG TG |
|  | R: CTT GTG TTC GCT CCC AGG CAT A |
| PSA | F: GAC CAA GTT CAT GCT GTG TGC |
|  | R: CCA CTC ACC TTTCCC CTC AAG |
| TMPRSS2 | F: CAA GTG CTC CAA CTC TGG GAT |
|  | R: AAC ACA CCG ATT CTC GTC CTC |
| S100P | F: AAG GAT GCC GTG GAT AAA TTG C |
|  | R: ACA CGA TGA ACT CAC TGA AGT C |
| KLK2 | F: TCA GAG CCT GCC AAG ATC AC |
|  | R: CAC AAG TGT CTT TAC CAC CTG T |

**Table S5. siRNAs.**

| siRNA | Sequence (5´to 3´) |
| --- | --- |
| siHAAO | CGAAGAGGGUGAAGAGGUATTUACCUCUUCACCCUCUUCGTT |
|  | GAGCAGUACAGAACAGGAATTUUCCUGUUCUGUACUGCUCTT |
| siFDPS | CCAGAAUUCAGAUGUUUAUTT |
|  | CCAUCAAUGAUGCUAACCUTT |

**Table S6. Risk genes**

| ENSG | Gene | Hazard Ratio | CI_Low | CI_Up | Pvalue | Beta |
| --- | --- | --- | --- | --- | --- | --- |
| ENSG00000196498 | NCOR2 | 2.904 | 1.348 | 6.258 | 0.0044 | 1.066089 |
| ENSG00000185591 | SP1 | 2.732 | 1.297 | 5.757 | 0.0059 | 1.005034 |
| ENSG00000078043 | PIAS2 | 0.36 | 0.167 | 0.775 | 0.0063 | -1.02165 |
| ENSG00000142453 | CARM1 | 2.668 | 1.269 | 5.61 | 0.0071 | 0.981329 |
| ENSG00000169375 | SIN3A | 2.56 | 1.217 | 5.382 | 0.0102 | 0.940007 |
| ENSG00000104081 | BMF | 2.452 | 1.163 | 5.17 | 0.0149 | 0.896904 |
| ENSG00000105229 | PIAS4 | 2.35 | 1.138 | 4.853 | 0.0173 | 0.854415 |
| ENSG00000169398 | PTK2 | 2.367 | 1.126 | 4.975 | 0.019 | 0.861623 |
| ENSG00000162882 | HAAO | 2.214 | 1.073 | 4.568 | 0.0271 | 0.794801 |
| ENSG00000082701 | GSK3B | 2.182 | 1.056 | 4.507 | 0.0307 | 0.780242 |
| ENSG00000173039 | RELA | 2.137 | 1.033 | 4.418 | 0.0364 | 0.759403 |
| ENSG00000001630 | CYP51A1 | 2.101 | 1.025 | 4.307 | 0.0384 | 0.742413 |
| ENSG00000103275 | UBE2I | 2.096 | 1.016 | 4.324 | 0.0407 | 0.740031 |
